# Different Mutations in a P-type ATPase Transporter in *Leishmania* Parasites are Associated with Cross-resistance to Two Leading Drugs by Distinct Mechanisms

**DOI:** 10.1371/journal.pntd.0005171

**Published:** 2016-12-02

**Authors:** Christopher Fernandez-Prada, Isabel M. Vincent, Marie-Christine Brotherton, Mathew Roberts, Gaétan Roy, Luis Rivas, Philippe Leprohon, Terry K. Smith, Marc Ouellette

**Affiliations:** 1 Centre de Recherche en Infectiologie du Centre de Recherche du CHU de Québec and Département de Microbiologie, Infectiologie et Immunologie, Faculté de Médecine, Université Laval, Québec, Québec, Canada; 2 Biomedical Sciences Research Complex (BSRC), Schools of Biology & Chemistry, The North Haugh, The University of St. Andrews, United Kingdom; 3 Centro de Investigaciones Biológicas (CSIC), Madrid, Spain; University of Antwerp, BELGIUM

## Abstract

*Leishmania infantum* is an etiological agent of the life-threatening visceral form of leishmaniasis. Liposomal amphotericin B (AmB) followed by a short administration of miltefosine (MF) is a drug combination effective for treating visceral leishmaniasis in endemic regions of India. Resistance to MF can be due to point mutations in the miltefosine transporter (MT). Here we show that mutations in MT are also observed in *Leishmania* AmB-resistant mutants. The MF-induced MT mutations, but not the AmB induced mutations in MT, alter the translocation/uptake of MF. Moreover, mutations in the MT selected by AmB or MF have a major impact on lipid species that is linked to cross-resistance between both drugs. These alterations include changes of specific phospholipids, some of which are enriched with cyclopropanated fatty acids, as well as an increase in inositolphosphoceramide species. Collectively these results provide evidence of the risk of cross-resistance emergence derived from current AmB-MF sequential or co-treatments for visceral leishmaniasis.

## Author Summary

Miltefosine and amphotericin B are two leading molecules in the fight against the vector-borne disease Leishmaniasis. We demonstrate here that different point mutations in a P-type ATPase could play an important role in resistance, not only to miltefosine, but also to amphotericin B. Furthermore, macrophage-infecting amastigotes that are resistant to one drug are cross-resistant to the other one. Our experimental findings demonstrate that one of the mechanisms driving miltefosine and amphotericin B resistance in *Leishmania* parasites involve major changes in a number of lipid species. In addition, we experimentally show that different mutations in the P-type ATPase participate in resistance against these two leading leishmanicidal agents with mechanistic differences. Miltefosine-induced mutations cause a defect in miltefosine uptake, but this mechanism of resistance is not observed for the amphotericin B-induced mutations. These results point to profound clinical implications as combination therapy involving these two drugs is being promoted for the treatment of visceral Leishmaniasis.

## Introduction

Protozoan parasites belonging to the *Leishmania* genus cause several vector-borne diseases collectively referred as leishmaniases. Currently, *Leishmania* species threaten *ca*. 350 million people in 88 countries worldwide [1]. Control measures primarily rely on prevention and chemotherapy (reviewed in [2]). The old-fashioned and toxic antimonial derivatives top the short list of registered compounds against *Leishmania* spp. In addition to their toxicity, pentavalent antimonials require long treatment schedules and are associated with resistance [1, 3]. Amphotericin B (AmB) liposomal formulations were introduced for the treatment of visceral leishmaniasis in antimonial-non-responsive regions of Bihar (India) [4]. Clinical resistance to AmB is rare [5] but a recent study in India has reported a *L*. *donovani* field strain resistant to AmB [6]. Another leishmanicidal drug introduced in the early 21^st^ century is the alkyl-phospholipid analogue miltefosine (MF). It was the first effective oral drug showing high cure rates in the treatment of several forms of leishmaniasis. However, since its registration in 2002, it has had increasing relapse rates and the emergence of drug resistance strains [7, 8].

None of these drugs have a well-defined mode of action against *Leishmania* spp. and primary protein drug targets are unlikely [9]. AmB seems to generate channel-like pores spanning the lipid bilayer by binding preferentially to ergosterol within the membranes, hence leading to cells death [10, 11]. Several reports suggest that MF is able to target lipid metabolism, in addition to glycosylphosphatidylinositol (GPI) anchor biosynthesis and signal transduction [12]. MF-treated parasites show an increase in phosphatidylethanolamine (PE) and *lyso*-phosphatidylcholine (PC) content in their membrane [13].

Both MF and AmB affect lipids in cellular membranes [11, 14], and resistance mechanisms seem to involve changes in lipids. AmB-resistance in *Leishmania* mainly implies changes in cell membrane fluidity (reviewed in [15]). The sterol content of *L*. *donovani* AmB-resistant promastigotes analysed by gas chromatography coupled to mass spectrometry (GC-MS) revealed an enrichment in cholesta-5,7,24-trien-3*β*-ol [11], which suggests a more fluid cellular surface. On the other hand, resistance to MF primarily implies a transport defect with inactivation of the P-type ATPase miltefosine transporter (MT), or of its regulatory subunit LdRos3, causing a decrease in the uptake of *lyso*-phospholipids [16–18]. A recent study has reported changes to the metabolism of lipids in *L*. *infantum* MF-resistant parasites [19], further supporting that MF influences fatty acid and/or sterol metabolism [20].

We report here that the MT is mutated in both MF and AmB resistant mutants. The mutations are associated with cross-resistance and correlate with major changes in membrane lipid composition. These modifications in lipid composition were analysed through a range of lipidomic approaches and we show that different mutations in MT trigger changes in lipid compositions leading to both MF and AmB resistance. These findings are of potential clinical relevance as the sequential treatment of liposomal AmB followed by a short 7-days administration of MF has been used against visceral leishmaniasis in India [21,22].

## Material and Methods

### *Leishmania* cultures

The *Leishmania infantum* (MHOM/MA/67/ITMAP-263) wild-type strain (Ldi263 wt) and the *in vitro* generated resistant mutants AmB1000.1 and MF200.5 [23, 24], which are respectively resistant to 1000 nM of AmB and 200 μM MF, were grown in SDM-79 medium at 25°C supplemented with 10% fetal bovine serum, 5 μg/mL of haemin at pH 7.0 with either 200 μM of MF (Miltefosine, Cayman Chem.) or 1 μM AmB (Amphotericin B solution, Sigma) for the mutant strains, and 40 μg/mL G418 (Geneticin, Gibco-BRL) for the episomal overexpressors. EC_50_ values were calculated based on dose-response curves analysed by non-linear regression with GraphPad Prism 5.01 software. An average of at least three independent biological replicates was performed for each determination. Statistical significance between the mock-transfected wild-type and the tested strains was evaluated by unpaired two-tailed t test.

### Whole genome sequencing for AmB1000.1 strain

Genomic DNA was prepared from a mid-log phase clonal culture of *L*. *infantum* 263 AmB1000.1. A paired-ends sequencing library was prepared with the Nextera DNA sample prep kit and sequenced on an Illumina MiSeq platform with 250-nucleotide paired-ends reads. An average genome coverage of over 50-fold was obtained for the mutant. This approach allowed us to identify point mutations when comparing with the reference genome sequence of *L*. *infantum JPCM5* (TriTrypDB version 8.0) [25] and *L*. *infantum* 263 wt [26]. Sequence reads were aligned to the *L*. *infantum* JPCM5 genome using the software bwa-mem [27]. The maximum number of mismatches was 4, the seed length was 32 and 2 mismatches were allowed within the seed. Read duplicates were marked using Picard (http://broadinstitute.github.io/picard) and we applied GATK for indel realignment and snp and indel discovery [28, 29] in *L*. *infantum* 263 AmB1000.1. PCR amplification and conventional DNA sequencing verified all putative point mutations detected by whole genome sequencing. Copy numbers variations were derived from read depth coverage by comparing the coverage of uniquely mapped reads between *L*. *infantum* 263 AMB1000.1 and *L*. *infantum* 263 wt along small non-overlapping genomic windows (5 kb) for the 36 chromosomes (normalized to the total number of uniquely-mapped reads for each strain) [30]. Several python and bash scripts were created to further analyze the data. The sequence data for *L*. *infantum* 263 AmB1000.1 is available at the EMBL European Nucleotide Archive (http://www.ebi.ac.uk/ena) under study accession ERP001815 and sample accession ERS176091.

### DNA constructs and transfection

The LinJ.13.1590 and LinJ.16.1240 genes of *L*. *infantum* (LinJ10_V5.0) were amplified from genomic DNA using compatible primer pairs and PCR fragments were ligated into pGEM T-easy (Promega, Madison, WI, USA) for confirming the quality of the insert by standard sequencing, and then cloned in the *Leishmania* expression vector pSP72αNeoα [31], which contains the gene neomycin phosphotransferase (NEO) as selectable marker in *Leishmania*. A total of 20 μg of plasmid DNA for episomal expression, either the empty vector (mock) or carrying the genes of interest, were transfected into *Leishmania* promastigotes by electroporation as described previously [31]. Selection was achieved in the presence of 40 μg/mL G418.

### Miltefosine uptake

Miltefosine uptake was performed as described previously [19]. Briefly, *Leishmania* parasites were incubated in the presence of MT-11C-BODIPY [32] for 1 h. Then fluorescence emission was recorded and used to calculate the moles of internalized MF analogue. An average of three independent biological replicates run in triplicate was performed. Statistical significance between the mock-transfected wild-type and the tested strains was evaluated by unpaired two-tailed t test.

### Macrophage infections

As previously published [9] macrophage infections and drug susceptibility assays were performed as following: PMA-differentiated THP-1 macrophages were infected with stationary-phase parasites at a ratio of 18:1, for 2 h at 37°C in a 5% CO_2_ atmosphere. Cells were maintained in drug-free medium for 48 h after which infected cells were either left untreated or treated for 96 h at 37°C. The number of infecting amastigotes per 100 macrophages was determined by examination of 100 macrophages per assay in two independent experiments run in triplicate, which allowed for calculating the parasitic index (P_Idx_) as the percentage of infected cells multiplied by the mean number of parasites per cell. Statistical significance between the mock-transfected wild-type and the tested strains was evaluated by unpaired two-tailed t test.

### Lipid extraction

Total lipids were extracted using a modified Bligh and Dyer method. Briefly, cells were washed with PBS, suspended in 100 μL PBS and transferred to a glass tube, 375 μL of 1:2 (v/v) CHCl_3_: MeOH added and vortexed. The samples were agitated vigorously for a further 10–15 min. The samples were now made biphasic by the addition of 125 μL of CHCl_3_, vortex and then 125 μL of H_2_O and vortexed again and centrifuged at 1000 g at RT for 5 min. The lower phase was transferred to a new glass vial and dried under nitrogen and stored at 4°C.

### Electrospray-mass spectrometry analysis

Total lipid extracts were dissolved in 15 μL of CHCl_3_: MeOH (1:2) and 15 μL of acetonitrile: iso-propanol: water (6:7:2) and analysed with a AB Sciex 4000 QTrap, a triple quadrupole mass spectrometer equipped with a nanoelectrospray source. Samples were delivered using a Nanomate interface in direct infusion mode (~125 nL/min). The lipid extracts were analysed in both positive and negative ion modes using a capillary voltage of 1.25 kV. MS/MS scanning (daughter, precursor and neutral loss scans) were performed using nitrogen as the collision gas with collision energies between 35–90 V. Each spectrum encompasses at least 50 repetitive scans.

Tandem mass spectra (MS/MS) were obtained with collision energies as previously described [33], phosphatidylinositol (PI)/ inositol-phosphoceramide (IPC) in negative ion mode, parent-ion scanning of m/z 241 (PI 32:0 internal standard); 35-65V, PE in negative ion mode, parent-ion scanning of m/z 196 (PE 28:0 internal standard); 20-35V, C19Δ parent ion mode scanning m/z 295. MS/MS daughter ion scanning was performed with collision energies between 35-90V. Assignment of phospholipid species is based upon a combination of survey, daughter, precursor and neutral loss scans, as well as previous assignments [33, 34]. The identity of phospholipid peaks was verified using the LIPID MAPS: Nature Lipidomics Gateway (www.lipidmaps.org). Accurate mass spectra were also acquired (± 2 ppm) on an Orbitrap MS to assist definitive assignment of C19Δ fatty acid containing PE species.

### Inositol analysis

For inositol analysis, a fixed number of cells from different strains were collected and lipids extracted as above. An internal standard of D_6_-*myo*-inositol was added to samples prior to hydrolysis by a strong acid (6M HCl, o/n at 110°C), derivatised with trimethylsilyl ethers and analysed by GC-MS, as published elsewhere [35]. *Myo*-inositol was quantified and the mean and standard deviations of three separate analyses were determined for IPC and PI inositol quantification, lipid samples underwent base hydrolysis 500 μL of concentrated ammonia and 50% propan-1-ol (1:1), followed by incubation for at least 5 h at 50°C. Upon drying under nitrogen and removal of traces of ammonia with 2 rounds of H_2_0/MeOH evaporation, the modified Bligh and Dyer method as described above was conducted to separate the IPC in the organic phase and inositol-phospho-glycerol derived from the PI, which had been deacylated into the aqueous phase. These two phases were dried down and processed for inositol content as described above.

### Identification and quantification of fatty acids

Full characterisation and quantification of the fatty acids by conversion to the corresponding fatty acid methyl esters (FAME) followed by GC-MS analysis was performed as previously described [36]. Briefly, mid-log cell-lines were spun down and triplicate aliquots equivalent to 10^8^ cells were transferred to 2 mL glass vessels and spiked with an internal standard fatty acid C17:0 (20 μL 1 mM) and dried under nitrogen. Fatty acids were released by base hydrolysis using 500 μL of concentrated ammonia and 50% propan-1-ol (1:1), followed by incubation for at least 5 h at 50°C. After cooling, the samples were evaporated to dryness with nitrogen and dried twice more from 200 μL of H_2_O/MeOH (1:1) to remove all traces of ammonia. The protonated fatty acids were extracted by partitioning between 500 μL of 20 mM HCl and 500 μL of ether, the aqueous phase is re-extracted with fresh ether (500 μL) and the combined ether phases were dried under nitrogen in a glass tube.

The fatty acids were converted to FAME by adding diazomethane (3 x 20 μL aliquots) to the dried residue on ice. After 30 min the samples were allowed to warm to RT and left to evaporate to dryness in a fume hood. The FAME products were dissolved in 10–20 μL dichloromethane and analysed by GC-MS by injection of 1–2 μL on a Agilent Technologies (GC-6890N, MS detector-5973) with a ZB-5 column (30 M x 25 mm x 25mm, Phenomenex), with a temperature program at 70°C for 10 min followed by a rising gradient to 220°C at 5°C /min and held at 220°C for a further 15 min. Mass spectra were acquired from 50–500 amu. The identity of FAMEs was carried out by comparison of the retention time and fragmentation pattern with a bacterial FAME standard that contains both C17Δ and C19Δ (Supelco).

### Sterol analysis

Lipid extractions of triplicate aliquots equivalent to 10^8^ cells were transferred to 2 mL glass vessels and dried down. The lipid extracts were dissolved in 20 μL dichloromethane and analysed by GC-MS by injection of 1 μL on a Agilent Technologies (GC-6890N, MS detector-5973), injector at 270°C with a ZB-50 column (15 mm x 32 mm id x 0.5 mm thickness, Phenomenex), injector at 270°C with a temperature program at 100°C for 1 min followed by a gradient to 200°C at 8°C /min and held at 200°C for a further 2 min followed by a second gradient to 300°C at 3°C /min and held for a further 15 min. Mass spectra were acquired from 50–550 atomic mass units. The identity of sterols was carried out by comparison of the retention time and fragmentation pattern with a range of standards purchased from Sigma and Materya.

## Results

### Whole genome sequencing of *L*. *infantum* aAmB1000.1 reveals a point mutation in the miltefosine transporter MT

The *in-vitro* selected resistant mutant *L*. *infantum* AmB1000.1 was previously characterized by means of a large-scale proteomic study [23], and here its genome was sequenced using paired-ends Illumina sequencing and compared to the one of its parent line. An average of 50-fold genome coverage was obtained for both the wild-type and the mutant. Read depth coverage analysis did not identify specific gene amplification or deletion in the mutant (S1 Dataset), although aneuploidy was observed for 6 chromosomes (S1 Table), a phenomenon often observed in drug resistant mutants [18, 37–39]. A search for point mutations revealed 18 homozygous single nucleotide polymorphisms (SNPs) in the AmB1000.1 mutant (S2 Table), 3 of which occurred within coding sequences (CDS) and caused an amino acid change (S2 Dataset). These occurred within the MT ORF (LinJ.13.1590), in gene LinJ.16.1240 coding for a hypothetical transmembrane protein and in LinJ.35.0520, a large proteophosphoglycan protein made up of a short 88-times repeated sequence which is often found mutated in our various sequencing screens. The latter gene was not studied further. The SNPs in MT and LinJ.16.1240 were confirmed by sequencing PCR fragments derived from AmB1000.1. An additional 470 heterozygous SNPs were also detected in the genome of AmB1000.1, 85 of them being in CDS and non-synonymous (S2 Table). Interestingly none of these occurred within the ORF coding for the regulatory subunit Ros3 that is necessary for the expression of a functional MT translocation machinery [17] (S3 Dataset).

### Mutations of the MT gene in AmB1000.1 modulate AmB resistance and is associated with cross-resistance to MF

**Fig 1:**
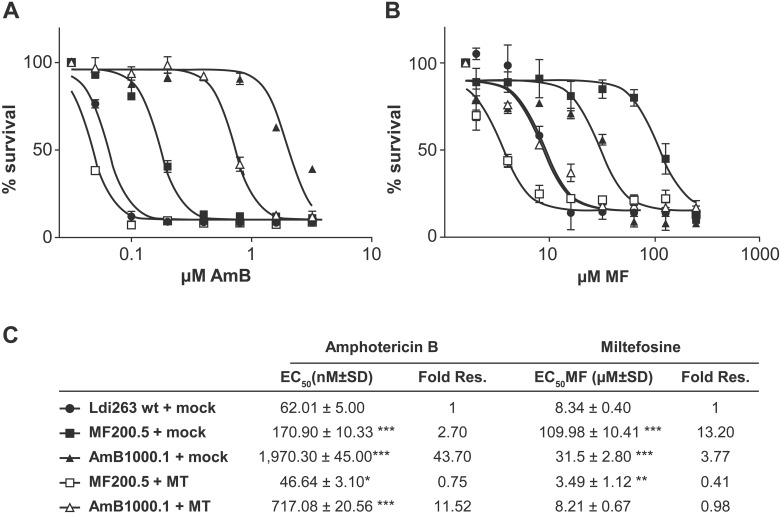
Role of mutations in the miltefosine transporter (MT) in miltefosine and amphotericin B cross-resistance. Dose-response curves with promastigotes in the presence of AmB **(A)** and MF **(B)** for Ldi263 wt (●); MF200.5 (■); AmB1000.1 (▲) mock-transfected parasites; MF200.5+MT (□); and AmB1000.1+MT (△) add-back cell lines over 72 h. An average of at least three independent biological replicates is shown, with error bars depicting the standard error of the mean. EC_50_ values were calculated from the dose-response curves after performing a nonlinear fitting with the Graphpad 5.0 software program **(C)**. Statistical significance between the mock-transfected wild-type and the rest of the strains was evaluated by unpaired two-tailed t-test (*p ≤ 0.*05*, **p ≤ 0.*01*, ***p ≤ 0.*001*).

We next tested whether mutations in the MT or the LinJ.16.1240 gene detected in AmB1000.1 directly contributed to AmB resistance. We also included the previously characterized MF resistant mutant *L*. *infantum* MF200.5 [24] with a known mutation in MT. Targeted sequencing of the MT in *L*. *infantum* MF200.5 confirmed the previously described G565R mutation, but also revealed two new mutations located at the very beginning of the gene and within a conserved domain (S1 Fig). The emergence of these new mutations is probably due to continuous culturing of the *L*. *infantum* MF200.5 mutant in the presence of high MF concentrations. The mutants AmB1000.1 and MF200.5 were highly resistant to AmB (Fig 1A and 1C) and MF respectively (Fig 1B and 1C). Remarkably, both mutants also showed MF/AmB cross-resistance, with mutant AmB1000.1 being 3.7 fold less sensitive to MF than wild-type parasites (Fig 1B and 1C) and MF200.5 being 2.7-fold cross-resistant to AmB (Fig 1A and 1C).

Transfection of the wild-type MT gene as part of an episomal vector in the *L*. *infantum* AmB1000.1 and MF200.5 lines abolished, as expected and previously described [17, 38], MF resistance in MF200.5 and interestingly in AmB1000.1 compared to mock-transfected parasites (Fig 1C). Surprisingly, transfection of the MT gene also reduced the AmB resistance to wild-type levels in the case of MF200.5 and partially reverted resistance to AmB by 4-fold for AmB1000.1 (Fig 1C). Overexpression of the wild-type copy of gene LinJ.16.1240 in *L*. *infantum* AmB1000.1 had no impact on the resistance phenotype against either AmB or MF (S2 Fig). This new role of MT in AmB resistance is not limited to *L*. *infantum* since selection for AmB resistance in *L*. *major* Friedlin similarly selected for a MT frameshift mutation in mutant *L*. *major* AmB1080.3 (S1 Fig). The mutant displayed a 2-fold cross-resistance to MF (S3A Fig) and transfection of a wild-type copy of the MT reduced not only resistance to MF, but also to AmB (3-fold) (S3B Fig).

**Fig 2:**
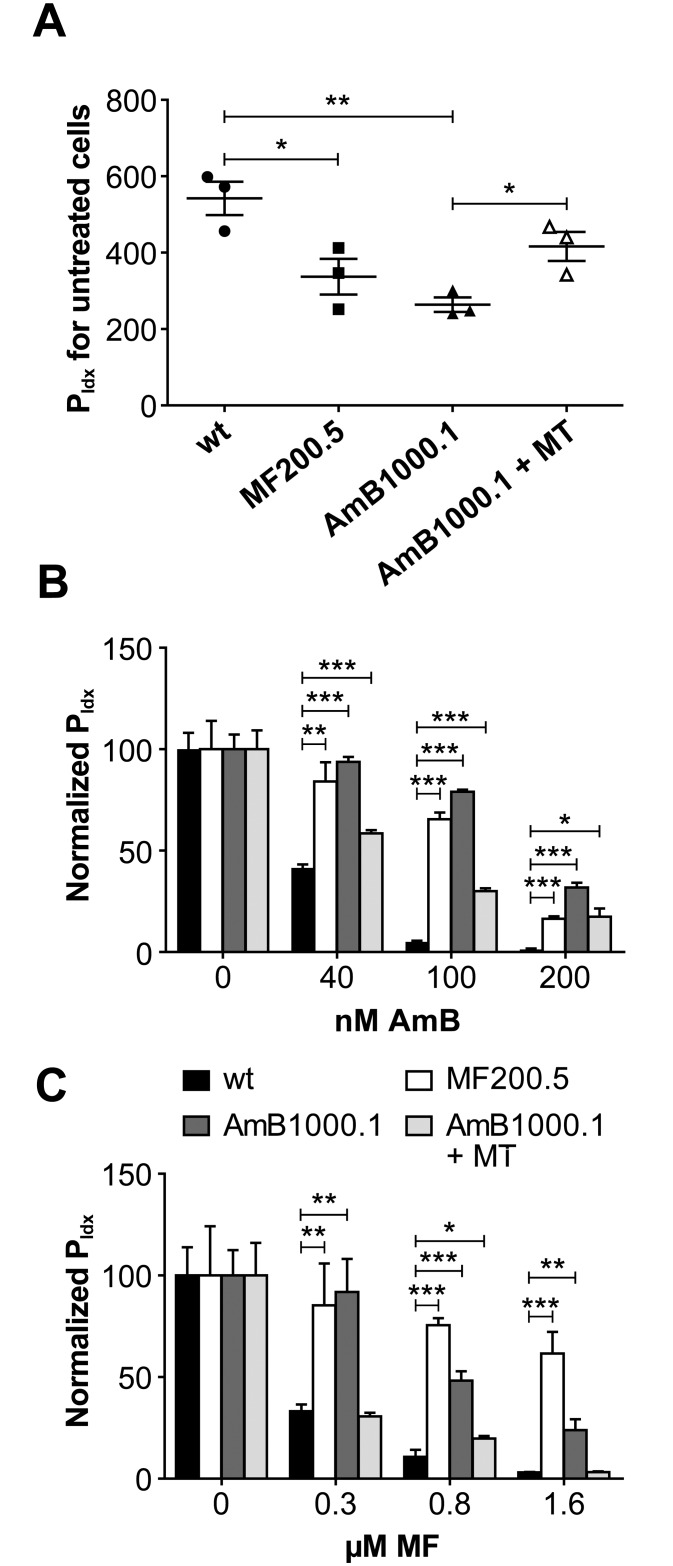
Infectivity of the different strains in THP-1-derived macrophages and intracellular drug resistance. **(A)** Infectivity of the different studied strains after 96h post-infection in the absence of drug treatment. The P_Idx_ represents the percentage of infected cells multiplied by the mean number of parasites per cell. Histogram showing normalized P_Idx_ dose-response effect after 96 h of exposition to increasing concentrations of AmB **(B)** and MF **(C)** for the strains studied in (A). Normalization represents the percent reduction of the total parasite burden compared to the non-treated infected control. Data are the mean ± S.D. of two independent experiments run in triplicate. Statistical significance between the mock-transfected wild-type and the rest of the strains was evaluated by unpaired two-tailed t test (*p ≤ 0.*05*, **p ≤ 0.*01*, ***p ≤ 0.*001*).

We also tested the survival of AmB1000.1 and MF200.5 in PMA-differentiated THP-1 macrophages and the role of MT in resistance in intracellular parasites. The P_Idx_, represented as the percentage of infected cells multiplied by the mean number of parasites per cell, of *L*. *infantum* wild-type parasites was 540 with a mean number of 6.5 amastigotes per macrophage, while the P_Idx_ for AmB1000.1 and MF200.5 were lowered by half including the number of amastigotes inside the infected macrophages (Fig 2A). The impaired infectivity was MT-related since there was a partial rescue of the phenotype in the AmB1000.1 MT add-back that reached a P_Idx_ of 420 with 5.5 parasites per macrophage (Fig 2A). The AmB1000.1 intracellular amastigotes were also resistant to AmB (Fig 2B) and maintained their cross-resistance to MF (Fig 2C) when compared to wild-type amastigotes. Similar results were found for mutant MF200.5 that maintained their cross-resistance to AmB inside macrophages (Fig 2B and 2C). Introducing a wild-type copy of the MT in AmB1000.1 led to a reduction in resistance levels to both antileishmanial agents (Fig 2B and 2C).

### The role of mutations in MT in miltefosine transport

**Fig 3:**
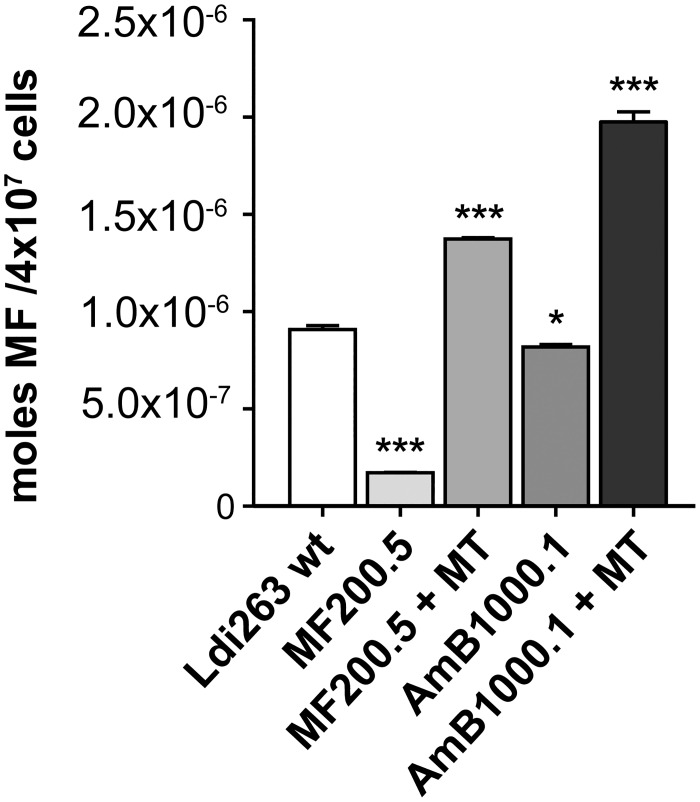
Miltefosine transport of drug resistant Leishmania. Histogram showing MF accumulation for the different strains generated and the wild-type mock-transfected line measured by fluorescence intensity of parasites treated with 5 μM MT-11C-BODIPY for 1 h. Untreated parasites were used to subtract the background noise. Data are the mean ± S.D. of three independent experiments run in triplicate. Statistical significance between the mock-transfected wild-type and the rest of the strains was evaluated by unpaired two-tailed t test (*p ≤ 0.*05*, ***p ≤ 0.*001*).

Mutations in MT are often associated with defects in MF transport [16, 17, 40]. Thus, mutations in MT in AmB1000.1 prompted us to probe the ability of the mutant to take up MF. We monitored the uptake of MT-11C-BODIPY, a fluorescent analogue of MF with *in vitro* leishmanicidal activity comparable to that of the original alkyl-phosphocholine [32]. The transport of MT-11C-BODIPY was greatly impaired in the MF200.5 mutant in comparison to the wild-type strain (Fig 3). In the mutant AmB1000.1 the decrease in accumulation of the fluorescent molecule was minimal but statistically significant (Fig 3). The introduction of an episomal copy of wild-type MT restored MF accumulation in the MF200.5 background whilst AmB1000.1 experienced a 2.6-fold increase in the amount of MF accumulated (Fig 3). This is in line with the MF re-sensitization observed for both mutants upon transfection of the rescue plasmid (Fig 1C).

### Changes in lipid-species content in amphotericin B and miltefosine resistant mutants harbouring mutations in the MT

Mutations in the MT can lead to cross-resistance to MF and AmB but it would appear that different mutations have different outcomes on MF transport (Fig 3). As MT translocates *lyso*-phospholipids, we hypothesized that changes in membrane lipid content triggered by mutations in the MT could be responsible for AmB/MF cross-resistance. The lipid/fatty acid composition of the mutants AmB1000.1 and MF200.5 were thus assessed and compared to those of wild-type and add-back cells.

Phospholipids (PLs) content in *L*. *infantum* 263 wild-type, MF200.5, AmB1000.1, and AmB1000.1 MT add-back parasites was analysed by electrospray ionisation-mass spectrometry (ES-MS). Negative ion ES-MS survey scans (600–1000 m/z) of total lipid extracts revealed significant changes in mutants AmB1000.1 (Table 1 and S4C Fig, upper panel) and MF200.5 (Table 1 and S4B Fig, upper panel) compared to wild-type parasites (S4A Fig, upper panel). We observed that several IPC lipid species from 34:0 to 38:0 were increased in both mutants (Table 1, S4A–S4C Fig upper panel). Transfection of the MT gene in AmB1000.1 brought back these IPC species closer to wild-type levels (Table 1). Two of the major PI species at 836 and 850 m/z were decreased in both mutants, probably as a result of increased IPC formation which requires PI. However, there was also a corresponding increase in the PI 42:8 species (934 m/z) (Table 1). Transfection of the MT gene in AmB1000.1 brought back the PI species closer to wild-type levels (Table 1). The PE species were relatively unchanged in MF200.5 and AmB1000.1 with the notable exceptions of a16:1/19Δ and 16:1/19Δ that were increased in both mutants (Table 1, S4 Fig upper panels). Again, the transfection of wild-type MT in AmB1000.1 brought back those lipid species closer to wild-type levels (Table 1, S4 Fig upper panels).

**Table 1:** Comparison of the lipid PI, IPC and PE species between WT and MF200.5, AmB1000.1 or AmB1000.1+MT. These comparisons are based upon parent-ion scanning of m/z 241 for PI and IPC (PI 32:0 was used as an internal standard); 35-65V and parent-ion scanning of m/z 196 for PE (PE 28:0 was used as an internal standard). When required parent-ion scanning of m/z 295 for C19Δand accurate mass assisted distinctions between PE species with the same nominal mass (S6 Fig and S3 Table). The symbols show relative increase or decrease relative to wild-type cells “+++, ++, +, =, -, --, ---”.

Observed mass (m/z)	Lipid species	MF200.5^a^	AmB1000.1^a^	AmB1000.1+MT^a^
**751**	IPC 32:1	=	=	=
**753**	IPC 32:0	=	=	=
**779**	IPC 34:1	++	++	+
**781**	IPC 34:0	+++	++	+
**807**	IPC 36:1	+++	++	+
**809**	IPC 36:0	+++	++	+
**833**	IPC 38:1	=	+	=
**835**	IPC 38:0	+	++	=
**861**	IPC 38:1	=	+	=
**836**	PI 34:1	-	--	--
**838**	PI 34:0	--	-	---
**848**	PI a-36:2	-	=	=
**850**	PI a-36:1	--	++	--
**852**	PI a-36:0	-	=	-
**860**	PI 36:3	=	=	++
**862**	PI 36:2	=	+	+
**864**	PI 36:1	=	+	=
**866**	PI 36:0	=	=	=
**892**	PI 38:1	--	-	+
**894**	PI 38:0	--	-	=
**934**	PI 42:8	+++	+++	=
**690**	PE 32:0	=	--	--
**698**	PE a-34:3	=	-	-
**700**	PE a-34:2	=	-	-
**712**	PE 34:3	+	=	=
**714**	PE 34:2/ PE a-16:1/19Δ	=/+	=/=	=/=
**716**	PE 34:1/ PE a-16:0/19Δ	=/+	=/+	=/=
**726**	PE a-36:3	+	=	=
**728**	PE a-36:2/16:1/19Δ	=/++	=/++	=/-
**742**	PE 36:2/ a-18:1/19Δ	=/=	=/+	=/-
**744**	PE 36:1/ a-18:0/19Δ	=/=	=/+	=/=
**766**	PE 38:4	=	=	=
**768**	PE 38:3	=	=	=
**770**	PE 38:2	=	=	=
**772**	PE 38:1	+	=	=

^a^Sometimes in Table 1 the lipid species share an observed mass (*e*.*g*. PE 34:1 and PE a-16:0/19Δ were detected at 716 (m/z)). While the presence of the first species may remain unaltered with respect to the wild-type, the second one may increase or decrease. The nomenclature =/+, =/++ and =/- was used in these cases.

The positive ion ES-MS survey scans (600–1000 *m*/*z*) of total lipid extracts derived from both mutants indicated slight variation in individual PC species. We also observed increased presence of ceramide species at 659 and 685 m/z for mutants MF200.5 (S4B Fig lower panel) and AmB1000.1 (S4C Fig lower panel). Introducing a wild-type copy of MT in AmB1000.1 reversed to some extent the observed differences (S4D Fig lower panel). The *lyso*-phospholipid content as determined by positive and negative ion survey scans (120–600 m/z) showed no significant differences between the WT, the resistant strains and the MT add back.

*Leishmania* PE species often contain the cyclopropyl fatty acid C19Δ [41, 42]. Several PE species were identified and confirmed by accurate mass spectrometry (S5 Fig, S3 Table), by parent ion ES-MS-MS of 295 m/z corresponding to the C19Δ fragment (S6A Fig) and by daughter ion fragmentation by ES-MS-MS (S6B–S6G Fig). The total fatty acid content, including C19Δ species, was determined in wild-type and resistant parasites. Derivatization of the total fatty acid content into their FAME enabled their quantification by GC-MS. Total ion chromatograms were obtained from mid log phase parasites for each strain and an example of the fatty acid distribution profile for wild-type *L*. *infantum* strain can be found in S7A and S7B Fig. All FAME species were identified and their relative percentages calculated (Table 2). Most FAMEs were similar across all strains, including the relative ratio of saturated and unsaturated fatty acids. However, both AmB1000.1 and MF200.5 mutants showed a ~4-fold increase in C19Δ. The second fatty-acid species altered significantly in both mutants corresponded to fatty acid C24:0, which was increased by 1.6-fold in AmB1000.1 and MF200.5 (Table 2). Episomal transfection of a wild-type copy of MT in AmB1000.1 restored both C19Δ and 24:0 fatty acids to near wild-type levels (Table 2).

**Table 2:** Total fatty acid content quantification (relative %). GC-MS was used to determine the fatty acid content of the different *L*. *infantum* strains, in comparison with wild-type parasites. S7 Fig includes an example for total ion chromatogram of derivatised fatty acids from lipid extracts of *L*. *infantum* 263 wild-type. Data are the mean of three independent experiments. Statistical significance between the mock-transfected wild-type and the rest of the strains was evaluated by unpaired two-tailed t test (***p ≤ 0.*001*).

Fatty acid	Retention time (min)	Relative quantification (%)			
		WT	AmB1000.1	AmB1000.1 +MT	MF200.5
14:0	31.2	2.1	2.3	2.6	2.2
16:1	35.3	0.2	0.1	0.1	0.1
16:0	35.6	2.8	2.9	2.9	2.8
18:3 n = 3	38.8	1.1	0.9	0.7	0.9
18:3 n = 6	38.9	1.1	1.0	0.9	0.9
18:2	39.1	28.9	27.9	28.1	28.4
18:1	39.2	24.6	20.3	24.7	19.6
18:0	39.7	19.8	19.0	18.7	19.2
C19Δ	40.8	0.7	4.1***	0.8	3.8***
20:4 n = 3	42.1	2.9	2.5	2.7	2.7
20:4 n = 6	42.2	2.1	2.1	2.0	2.0
20:3	42.4	0.5	0.2	0.3	0.5
20:2	42.6	3.8	4.0	3.9	4.2
20:1	42.8	0.6	0.4	0.5	0.5
20:0	42.9	0.2	0.1	0.1	0.1
22:6 n-3	44.1	1.8	0.9	1.4	1.7
22:5 n-3	44.2	2.0	1.8	1.7	1.9
22:2	44.7	1.6	1.2	1.6	1.4
24:5 n-6	46.1	1.8	1.9	1.7	1.8
24:4 n-6	46.4	2.7	2.8	2.9	3.5
24:0	48.9	1.7	2.8***	1.6	2.7***
**% Saturated**		26.6	27.1	25.7	27.0
**% Unsaturated**		73.4	72.9	74.3	73.0

Sphingolipids (SL) are major component of *Leishmania* membranes [41] and *Leishmania* do not process the *de novo* biosynthetic pathways for neither sphingomyelin, nor complex glyco-SLs [41]. The overall relative abundance of IPCs and PIs, as well as the total amount of lipid-inositol, were thus determined in mutants AmB1000.1 and MF200.5 and compared to those of wild-type parasites (Table 3). *L*. *infantum* 263 wild-type presented a relative distribution of 39% IPC and 61% PI of their total lipid containing inositol species. AmB1000.1 and MF200.5 mutants showed a significant increase in IPC formation, with percentages of 51%-53% for IPC and 49%-47% for PI, respectively. Interestingly, introduction of the wild-type copy of the MT in the AmB1000.1 mutant resulted in a shift back of the IPC-PI percentages to 45% -55%, values closer to wild-type parasites. The AmB and MF mutants also showed a significant increase in total inositol containing lipids, highlighting the significant increase in IPC content within these cells. The total inositol-lipid content was returned to wild-type like levels in the add-back line (Table 3).

**Table 3:** IPC/PI ratio and total inositol quantification relative to the wild-type strain. Data are the mean ± s.d. of two independent experiments run in triplicate. Statistical significance of INO between the mock-transfected wild-type and the rest of the strains was evaluated by unpaired two-tailed t test (*p ≤ 0.*05*, **p ≤ 0.*01*, ***p ≤ 0.*001*).

		Abundance relative to total INO (%)	Total INO relative to WT (%)	Abundance relative to WT (%)
**Ldi263 wt**	IPC	39.0 ± 1.4	---	---
	PI	61.0 ± 1.4		---
**AmB1000.1**	IPC	51.4 ± 1.0***	127.3 ± 6.0**	167.5 ± 2.6***
	PI	48.6 ± 1.0***		102.5 ± 2.6
**AmB1000.1+MT**	IPC	44.9 ± 1.1***	106.2 ± 1.7*	121.1 ± 1.7**
	PI	55.1 ± 1.1***		96.0 ± 1.7
**MF200.5**	IPC	52.8 ± 1.1***	116.1 ± 4.6*	156.5 ± 2.4**
	PI	47.2 ± 1.1***		90.5 ± 2.4*

To complete our analysis of lipids, and because of the suspected mode of action on AmB, we also measured sterols by GS-MS in AmB1000.1, AmB1000.1+MT, MF200.5 and wild-type cells (S8 Fig). Levels of sterols in wild-type cells (Table 4) were similar to what observed in other species [43, 44]. The level of ergosterol and 5-dehydroepisterol were greatly decreased in AmB1000.1 while 4-methyl-8,24-cholestadienol was strongly increased (S8 Fig; Table 4). Surprisingly ergosterol was markedly decreased in MF200.5 as well, but episterol was now the dominant sterol (S8 Fig; Table 4). In contrast to other lipid species (Tables 1, 2 and 3) transfection of wild-type MT in AmB1000.1 did not modify the distribution of sterols (S8 Fig, Table 4).

**Table 4:** GC-MS analysis of sterols in Ldi263, MF200.5, AmB1001.1 and AmB1000.1+MT. Data are the mean ± s.d. of three independent experiments. Relative percentages based upon peak areas. TIC of chromatogram 39.50–43.50 min shown in S8 Fig. ND-not detected.

Label	Molecular Ion (*m/z*)	Annotation	Relative Percentages			
			Ldi263	MF200.5	AmB1001.1	AmB1000.1+MT
**1**	386	Cholesterol	11.0±1.8	10.7±1.6	5.3±0.7	5.2±0.4
**2**	396	5-dehydroepisterol	68.8±4.2	19.6±2.0	1.3±0.2	1.3±0.1
**3**	396.	Ergosterol	7.8±0.6	trace	trace	trace
**4**	382	Cholesta-5,7,24-trienol	12.2±0.8	0.7±0.2	13.9±1.2	12.8±1.5
**5**	366	Episterol	ND	68.2±3.9	ND	ND
**6**	412	14-methyl-fecosterol	ND	0.8±0.1	6.6±0.2	7.0±0.2
**7**	384	Zymosterol	ND	ND	1.2±0.1	1.1±0.1
**8**	384	cholesta-7,24-dienol	ND	ND	7.6±0.3	6.7±0.5
**9**	398	4-methyl-8,24-cholestadienol	ND	ND	73.1±6.0	65.9±4.7

## Discussion

Current clinical policies against visceral leishmaniasis in the endemic region of Bihar in India support the use of sequential treatments relying on administration of liposomal AmB followed by a short 7-days administration of MF [21, 22]. Resistance is not a current threat for AmB clinical use [5] although *L*. *donovani* field strains unresponsive to AmB have been reported [6]. Drug combination treatments involve shorter dosing schedules, which increases compliance and are less prone to select drug-resistant parasites compared to classical single-drug therapies [8, 45]. However, the existence of shared resistance mechanisms between two of the main leishmanicidal agents could lead to treatment failure and emergence of new refractory parasitic populations. Indeed, it has been recently reported that *L*. *donovani* can become resistant to drug combinations, including the combination of AmB/MF, and that the multi-resistant phenotypes are maintained in amastigotes [46]. Similarly, the characterization of several *L*. *donovani* field isolates revealed that susceptibility profiles against MF and AmB were positively correlated, thus identifying a risk for cross-resistance [46]. In this study we demonstrate that cells selected for AmB can be cross-resistant to MF and the reverse is also true. We also provide evidence for one pathway of cross-resistance through lipid content modifications, which is seemingly linked to mutations in the MT. This cross-resistance should lead to careful considerations when sequential treatments are considered in endemic regions [21, 22] especially because resistance is observed also for intracellular parasites (Fig 2).

The mutations in the MT gene of *L*. *infantum* MF200.5 and *L*. *major* AmB1080.3 lines are predicted to be in conserved domains of the MT (S1 Fig), whilst the G433S substitution in the MT of *L*. *infantum* AmB1000.1 is located nearby the DKTGTLT motif of the ATPase phosphorylation domain [47]. The lack of a structure for MT renders it difficult to predict the impact of this mutation on the function of the transporter, but lipid composition is altered and reintroducing an episomal copy of MT revert in part the mutation phenotype (Tables 1, 2 and 3). Reintroduction of a wild-type copy of MT also reverted resistance to both MF and AmB (Fig 1), and intracellular survival (Fig 2). Links between mutations in P-type ATPase and the import of different phospholipid species has been reported in mammalian cells [48–50]. Mutation in MT in MF200.5 is correlated with a decrease in MF uptake (Fig 3) and this likely contributes to MF resistance. In contrast, the uptake of MF is only minimally changed in AmB1000.1, suggesting that the mutation in MT may lead to MF cross-resistance by another mechanism. One possibility is that the changes in lipid composition in AmB1000.1 allow for a higher accumulation of MF within its membrane. Indeed, the AmB1000.1 mutant expressing wild-type MT through episomes had a susceptibility to MF identical to that of wild-type parasites (Fig 1) despite accumulating twice as much MF (Fig 3). Our accumulation experiments cannot distinguish between genuine intracellular uptake from accumulation of the drug at the level of the plasma membrane. It is thus possible that lipid-related compensatory mechanisms developed by AmB1000.1 may lead to less MF intracellular translocation. While the regulatory subunit Ros3 [7] is not mutated in AmB1000.1, we cannot exclude an impaired expression and its contribution, if any, to AmB-MF cross-resistance remains to be clarified.

Overexpressing a wild-type version of the MT in AmB1000.1 did not totally reverted its resistance to AmB and other mechanisms, such as the upregulation of proteins implicated in protection against drug-induced oxidants [23], should complement the protective effect achieved through changes in cellular-membrane lipids. For example, the level of sterols is altered in AmB1000.1 and this is independent of the MT (Table 4). This change in sterol may contribute to resistance but further work would be required to isolate the genes involved in those sterol changes. It is salient to point out that AmB1000.1 shows aneuploidy for 6 chromosomes (S1 Table) and that many SNPs were detected in its genome, 85 of which were in coding sequences (S2 Table, S2 and S3 Datasets), and some may have a role in AmB resistance. Besides resistance, MT-mediated lipid changes may also impact on parasite-macrophage interactions. As observed here for *L*. *infantum* AmB1000.1 and MF200.5, AmB-resistant [11, 51] and MF-resistant cells [52] had previously been reported to be less infective, suggesting that parasites with mutations in the MT may be selected against in the absence of drug pressure. This is not always the case however, as MF-resistant *L*. *major* [52] or *L*. *amazonensis* [53] with mutations in the MT did not show reduced infectivity, and the potential for resistance to multiple drugs by a single point mutation remains real.

Several studies have pointed to modifications in lipid metabolism as a major factor for both AmB [11, 54] and MF resistance in *Leishmania* [13, 14, 19, 20, 40]. Among the lipid species whose abundances were similarly altered in the AmB1000.1 and MF200.5 mutants is the noteworthy increase of IPC, PI and 19Δ-containing PE species. Interestingly, preliminary lipid quantification experiments with the *L*. *major* AmB1080.3 mutant revealed variations in both negative and positive ion ES-MS survey scans (S3C Fig) similar to those found for mutant *L*. *infantum* AmB1000.1 (S4C Fig). A recent study found that *L*. *donovani* parasites exposed to MF exhibit increased levels of three PE species [55], which support our findings regarding the increased levels of 19Δ-containing PE for both mutants. Interestingly, disruption of the LdMT-LdRos3 complex in *L*. *donovani* was shown to affect the asymmetry of membrane lipids and resulted in an increased exposure of PE to the exoplasmic leaflet of the plasma membrane [56]. However, in contrast to our resistant selected lines, these disrupted lines did not show significant differences in the total amount of PE and PI [56].

Analysis of FAMEs also confirmed the significant increase of C19Δ for both AmB1000.1 and MF200.5 mutants (Table 2). It has been reported that C19Δ is not abundant in wild-type *L*. *infantum* [36], and since its formation requires a high energy cost [57] it should provide important survival gain to the parasite. Interestingly, studies in *E*. *coli* have shown how cyclopropane fatty acids (CFAS)-mediated membrane modifications protect against environmental stresses (temperature, pH, salt concentration, etc.) [58. 59]. However, the impact of CFAS on the fluidity of the lipid bilayer is unclear, some studies supporting that defect in CFAS would result in a decrease of membrane fluidity [60] but others suggesting an enhanced fluidity [61]. In addition to C19Δ, lignoceric acid 24:0 was also increased in both AmB1000.1 and MF200.5 mutants. Lignoceric acid was previously detected in MF-resistant *L*.*-donovani* promastigotes but not in wild-type cells [20].

The content of SLs was also found to be modified in resistant parasites, with IPC being greatly increased in both mutants (Table 3). This correlates well with the enhanced levels of d16:0/18:0-IPC identified in MF-treated *L*. *donovani* [55]. Complex SLs like IPC are found in the outer leaflet of plasma membranes together with sterols and contribute to the overall plasma-membrane fluidity [62]. Moreover, changes in IPC-PI levels, most probably due to PI being the precursor for IPC formation, may also act as a compensatory effect due to the lack of optimal fatty acid containing diacyl-phospholipids secondary to defective MT activity. This in turn may also impact upon the PI species available for GPI anchor biosynthesis and usage.

While MT point mutations play a direct role in MF-resistant strains, it seems they can also induce lipid-content modifications in AmB resistant cells that favour cross-resistance to MF, most likely by increasing the ability of the parasite to accumulate MF in its membrane rather than hindering its uptake. This would suggest that resistance to AmB but also cross-resistance to MF is much likely related to changes in lipid composition and possibly in the intracellular routing of MF. Different mutations in MT have thus diverse functional consequences on the transport of MF (Fig 3). These results reinforce and expand the knowledge on the complex nature of AmB resistance in *Leishmania* parasites [23, 46, 63], by including a new and unexpected character that also triggers MF cross-resistance. This could have significant impact on the control of this neglected disease.

## Supporting Information

S1 Fig Graphical representation of the different mutations identified in MT for three Leishmania drug resistant strains. The diagram includes the different conserved protein domains identified for the MT (GenBank: AAQ82704.1).
(TIF)

S2 Fig Effect of the transfection of an episomal copy of the wild-type gene LinJ.16.1240 in the AmB1000.1 mutant background. (A) EC_50_ determination curves in the presence of amphotericin B for Ldi263 wt (62.01 ± 5.00 nM), AmB1000.1 (1.97 ± 0.04 μM) and AmB1000.1+LinJ.16.1240 (1.89 ± 0.11 μM) cell lines over 72 h. (B) EC_50_ determination curves in the presence of miltefosine for Ldi263 wt (8.34 ± 0.40 μM), AmB1000.1 (31.50 ± 2.80 μM) and AmB1000.1+LinJ.16.1240 (29.89 ± 3.03 μM) cell lines over 72 h. An average of at least three independent biological replicates is shown, with error bars depicting the standard error of the mean. EC_50_ values were determined by means of Graphpad Prism5 using non-linear regression analysis.
(TIF)

S3 Fig Preliminary study for the characterization of mutant L. major Friedlin AmB1080.3. (A) EC_50_ determination curves in the presence of miltefosine for LmF wt+mock (9.05 ± 0.89 μM), AmB1080.3+mock (54.84 ± 5.30 μM) and AmB1080.3+MT (4.43 ± 0.62 μM) cell lines over 72 h. (B) EC_50_ determination curves in the presence of amphotericin B for LmF wt+mock (0.16 μM ± 0.01), AmB1080.3+mock (1.50 ± 0.07 μM) and AmB1080.3+MT (0.60 ± 0.02 μM) cell lines over 72 h. An average of at least three independent biological replicates is shown, with error bars depicting the standard error of the mean. EC_50_ values were determined by means of Graphpad Prism5 using non-linear regression analysis. (C) Negative (upper panel) and Positive (lower panel) ion ES-MS survey scans (600–1000 m/z) of total lipid extracts from mutant L. major Friedlin AmB1080. PEs: phosphatidylethanolamines, IPCs: inositol-phosphoceramides and PIs: phosphatidylinositols. An example of one of three independent biological replicates (which showed similar profiles) is shown.
(TIF)

S4 Fig Identification of abundance changes to the membrane lipids in AmB- and MF-resistant parasites. Negative ion ES-MS survey scans (600–1000 m/z) of total lipid extracts from Ldi263 wt (A upper panel), MF200.5 (B upper panel), AmB1000.1 (C upper panel) and AmB1000.1+MT (D upper panel). Positive ion ES-MS survey scans (600–1000 m/z) of total lipid extracts Ldi263 wt (A lower panel), MF200.5 (B lower panel), AmB1000.1 (C lower panel) and AmB1000.1+MT (D lower panel). PEs: phosphatidylethanolamines, IPCs: inositolphosphoceramides and PIs: phosphatidylinositols. The different species identified in the ES-MS surveys are detailed in the Supplementary S4 Table. An example of one representative of three independent biological replicates is shown.
(TIF)

S5 Fig High resolution orbitrap mass spectrometry survey scans (600–1000 m/z) of total lipid extracts from Ldi263 wt; negative ion mode (A), positive ion mode (B). (PDF)

S6 Fig Characterisation of the PE species containing C19Δ in total lipid extracts from Ldi263 wt. (A) ES-MS/MS parents of 295 m/z (C19Δ acyl fragment) in negative mode (600–1000 m/z). (B-G) are daughter ion ESI-MS/MS spectra of ions identified in S6A Fig: 716, 730, 744, 774, 788, 802 m/z respectively.
(PDF)

S7 Fig Example of total ion chromatogram of derivatised fatty acids from lipid extracts of L. *infantum* 263 wild-type. (A) Chromatogram including all the FAMES species with retention times spanning from 26.00 to 50.00 min for mid log phase parasites for each strain detailed in Table 1. (B) Magnification of the chromatogram for the identification of the spectral peak with a retention time of ∼40 min corresponding to C19Δ. The identity of C19Δ FAME was confirmed by retention time and spectral comparison with bacterial FAME standards, which includes C19Δ.
(PDF)

S8 Fig GC-MS Sterol Analysis of *Leishmania* strains. TIC of chromatogram 39.50–43.50 min for (A) Ldi263, (B) MF200.5, (C) AmB1000.1, (D) AmB1000.1+MT. Numbered peaks refer to Table 4 for identification.
(TIF)

S1 Table Estimated ploidy for Ldi263AmB1000.1 and its WT parent (Ldi263WT). (PDF)

S2 Table SNPs deduced from the sequencing of LdiAmB1000.1 and absent from the sequencing of its Ldi263WT parent. (PDF)

S3 Table Mass spectrometric analysis of phosphatidylethanolamine species containing cyclopropyl fatty acid in *Leishmania infantum*. (PDF)

S1 Dataset InDels. (PDF)

S2 Dataset AmB_SNPs-InCDS-NoSyn-Homozygous. (PDF)

S3 Dataset AmB_SNPs-InCDS-NoSyn-Heterozygous. (PDF)
