# Supplementary figures and images for: Different Mutations in a P-type ATPase Transporter in Leishmania Parasites are Associated with Cross-resistance to Two Leading Drugs by Distinct Mechanisms
Source: PLoS Negl Trop Dis. 2016 Dec 2;10(12):e0005171. doi: 10.1371/journal.pntd.0005171 (PMC5135041; doi:10.1371/journal.pntd.0005171)

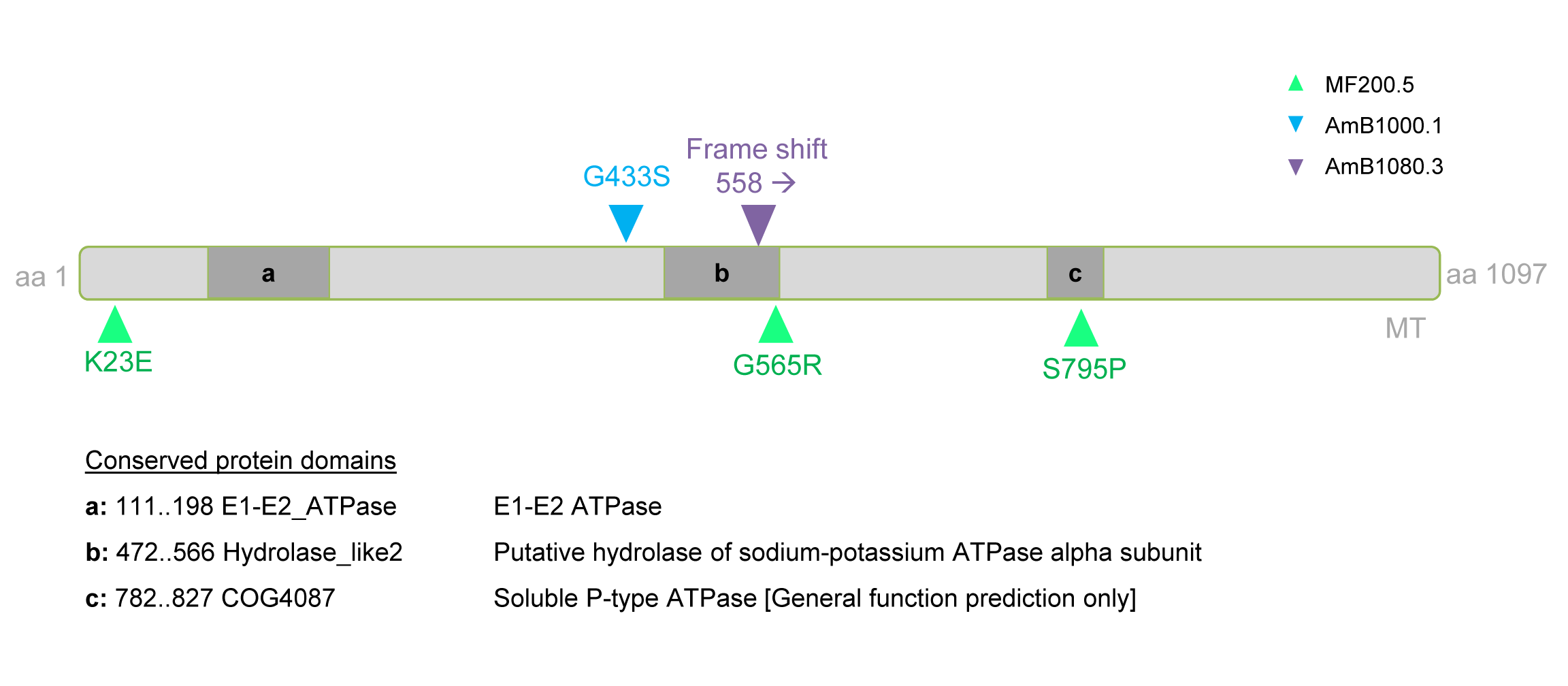

Supplement: S1 Fig — The diagram includes the different conserved protein domains identified for the MT (GenBank: AAQ82704.1). (TIF) [file pntd.0005171.s001.tif]

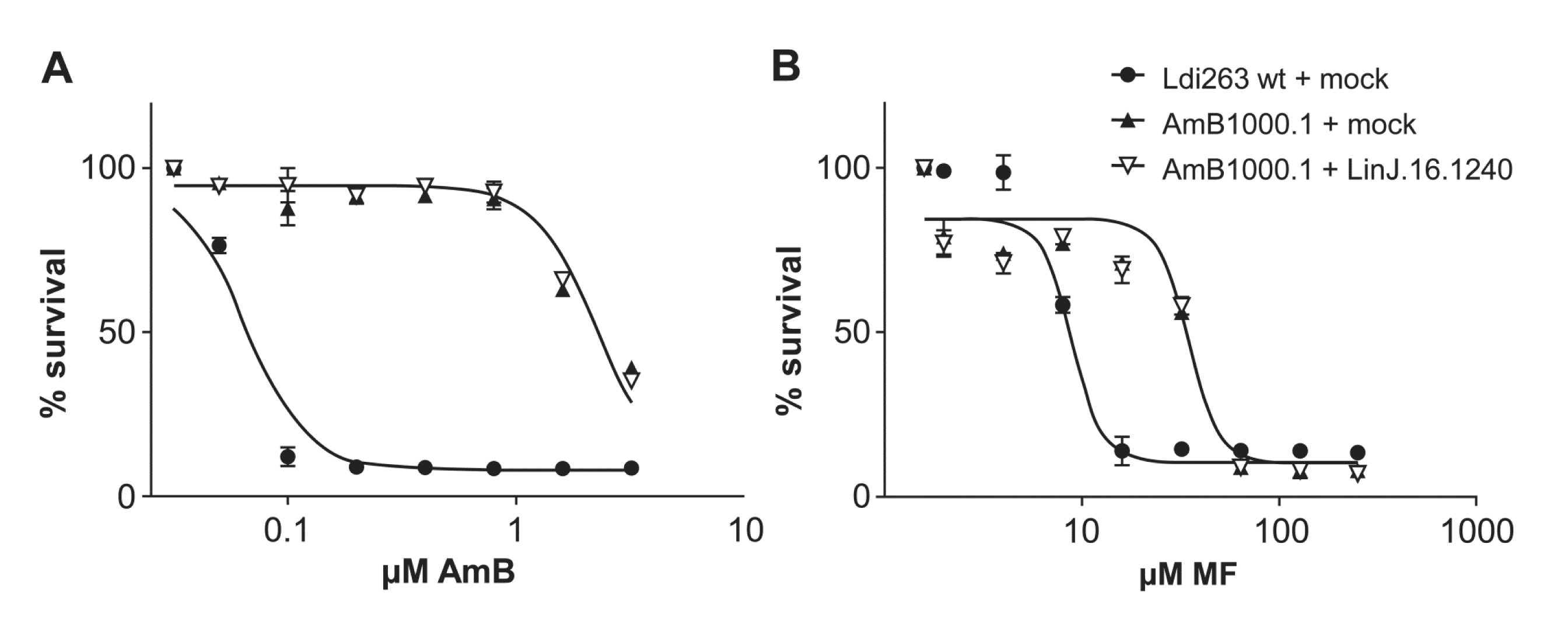

Supplement: S2 Fig — (A) EC50 determination curves in the presence of amphotericin B for Ldi263 wt (62.01 ± 5.00 nM), AmB1000.1 (1.97 ± 0.04 μM) and AmB1000.1+LinJ.16.1240 (1.89 ± 0.11 μM) cell lines over 72 h. (B) EC50 determination curves in the presence of miltefosine for Ldi263 wt (8.34 ± 0.40 μM), AmB1000.1 (31.50 ± 2.80 μM) and AmB1000.1+LinJ.16.1240 (29.89 ± 3.03 μM) cell lines over 72 h. An average of at least three independent biological replicates is shown, with error bars depicting the standard error of the mean. EC50 values were determined by means of Graphpad Prism5 using non-linear regression analysis. (TIF) [file pntd.0005171.s002.tif]

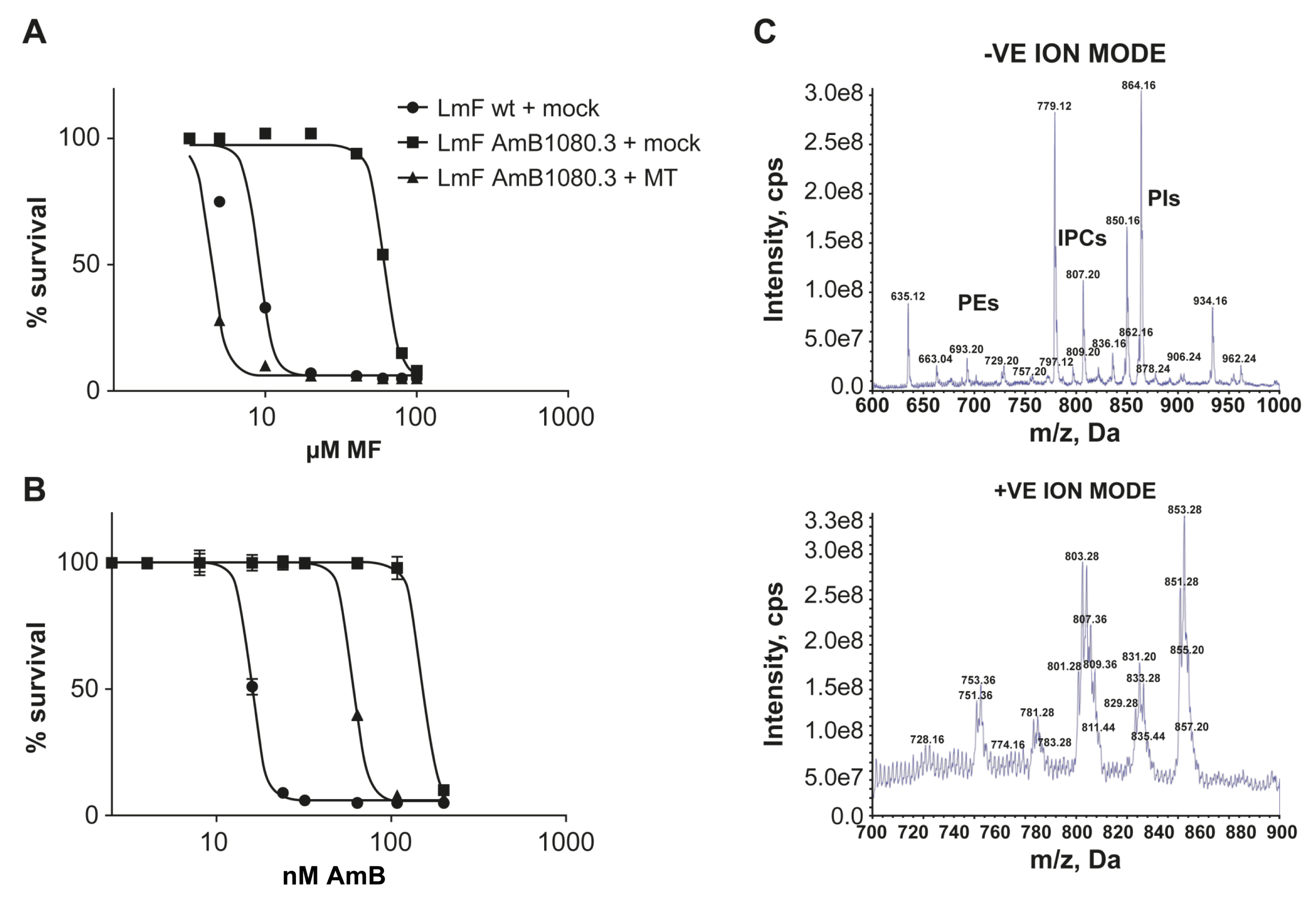

Supplement: S3 Fig — (A) EC50 determination curves in the presence of miltefosine for LmF wt+mock (9.05 ± 0.89 μM), AmB1080.3+mock (54.84 ± 5.30 μM) and AmB1080.3+MT (4.43 ± 0.62 μM) cell lines over 72 h. (B) EC50 determination curves in the presence of amphotericin B for LmF wt+mock (0.16 μM ± 0.01), AmB1080.3+mock (1.50 ± 0.07 μM) and AmB1080.3+MT (0.60 ± 0.02 μM) cell lines over 72 h. An average of at least three independent biological replicates is shown, with error bars depicting the standard error of the mean. EC50 values were determined by means of Graphpad Prism5 using non-linear regression analysis. (C) Negative (upper panel) and Positive (lower panel) ion ES-MS survey scans (600–1000 m/z) of total lipid extracts from mutant L. major Friedlin AmB1080. PEs: phosphatidylethanolamines, IPCs: inositol-phosphoceramides and PIs: phosphatidylinositols. An example of one of three independent biological replicates (which showed similar profiles) is shown. (TIF) [file pntd.0005171.s003.tif]

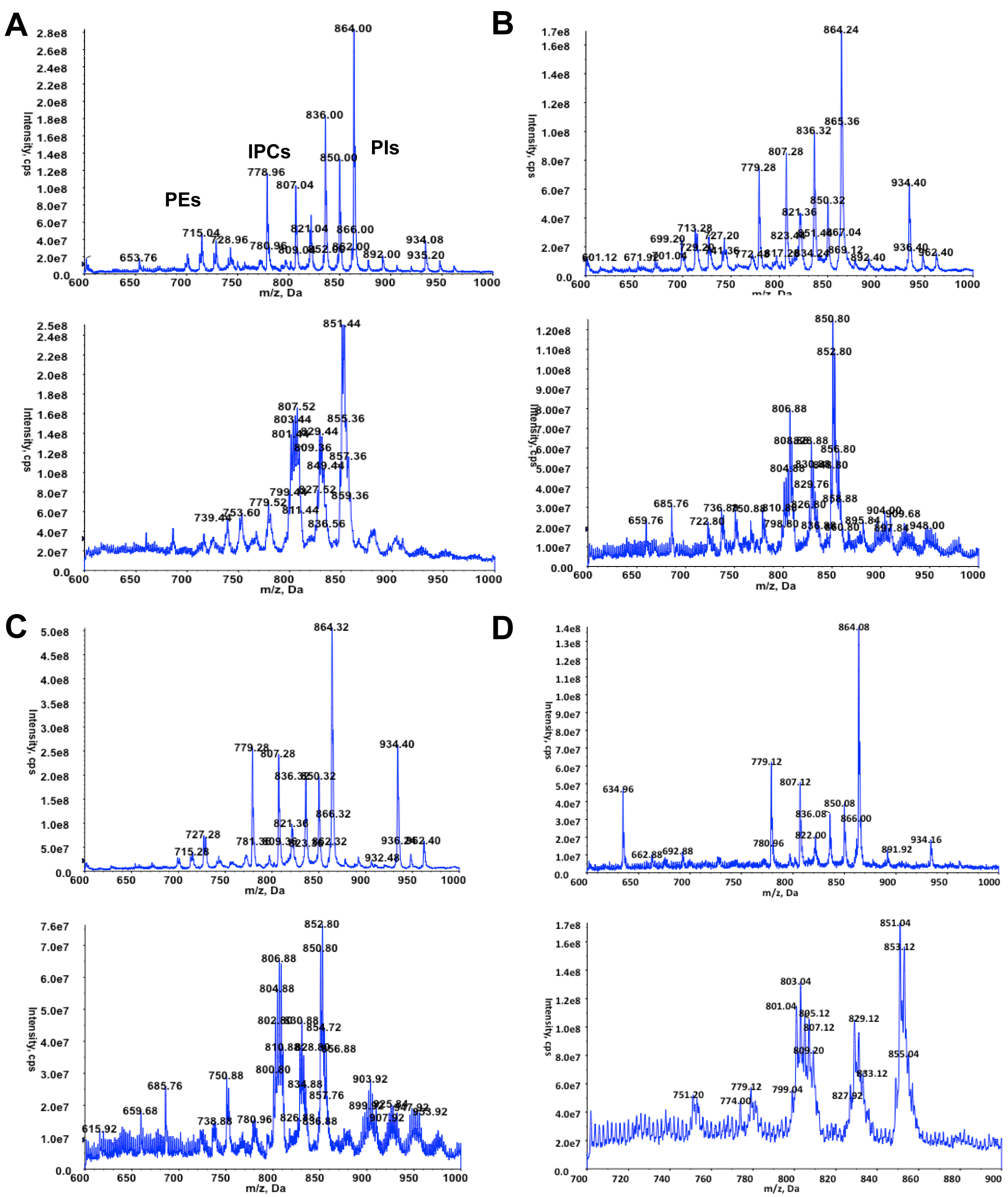

Supplement: S4 Fig — Negative ion ES-MS survey scans (600–1000 m/z) of total lipid extracts from Ldi263 wt (A upper panel), MF200.5 (B upper panel), AmB1000.1 (C upper panel) and AmB1000.1+MT (D upper panel). Positive ion ES-MS survey scans (600–1000 m/z) of total lipid extracts Ldi263 wt (A lower panel), MF200.5 (B lower panel), AmB1000.1 (C lower panel) and AmB1000.1+MT (D lower panel). PEs: phosphatidylethanolamines, IPCs: inositolphosphoceramides and PIs: phosphatidylinositols. The different species identified in the ES-MS surveys are detailed in the Supplementary S4 Table. An example of one representative of three independent biological replicates is shown. (TIF) [file pntd.0005171.s004.tif]

**A**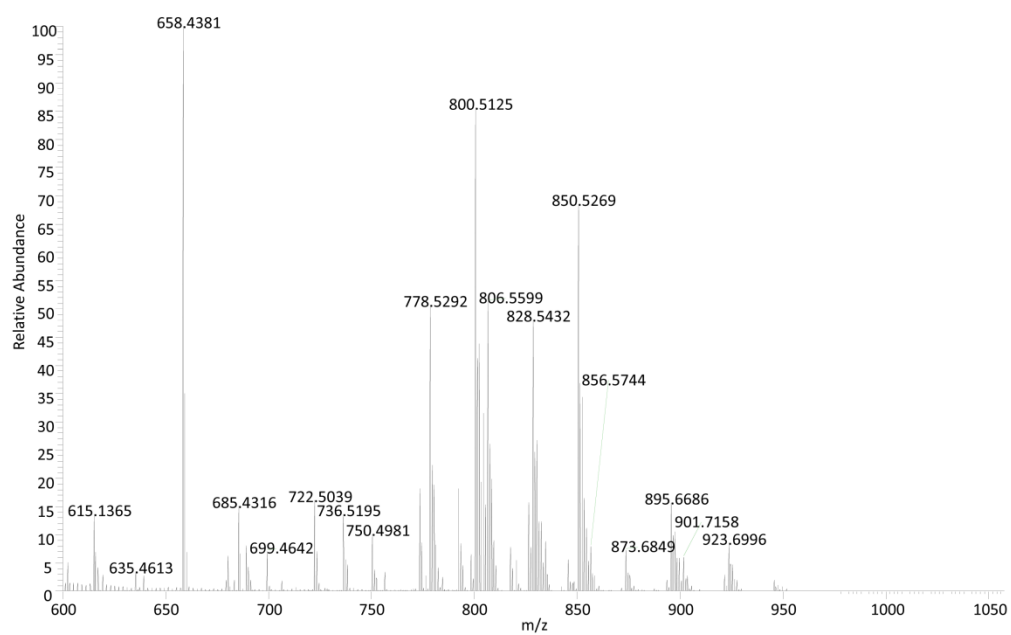**B**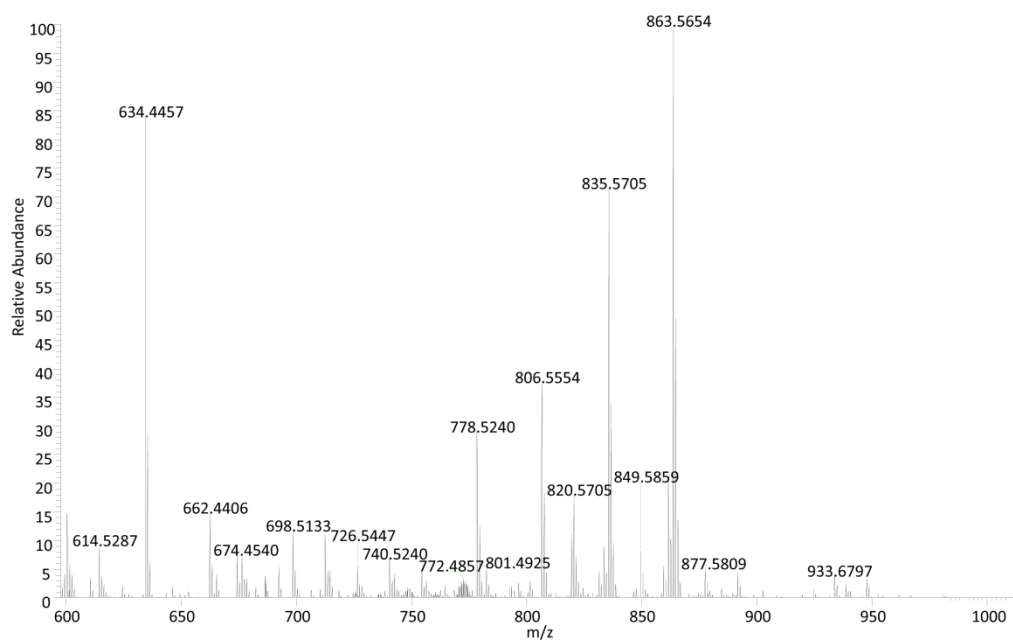

Supplement: S5 Fig — (PDF) [file pntd.0005171.s005.pdf]

**A**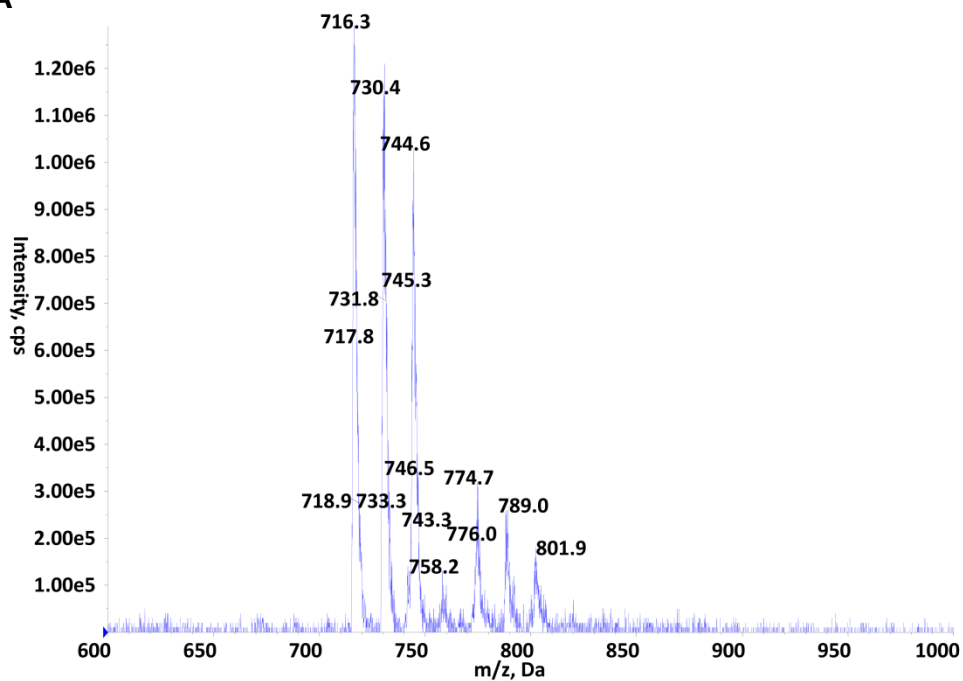**B**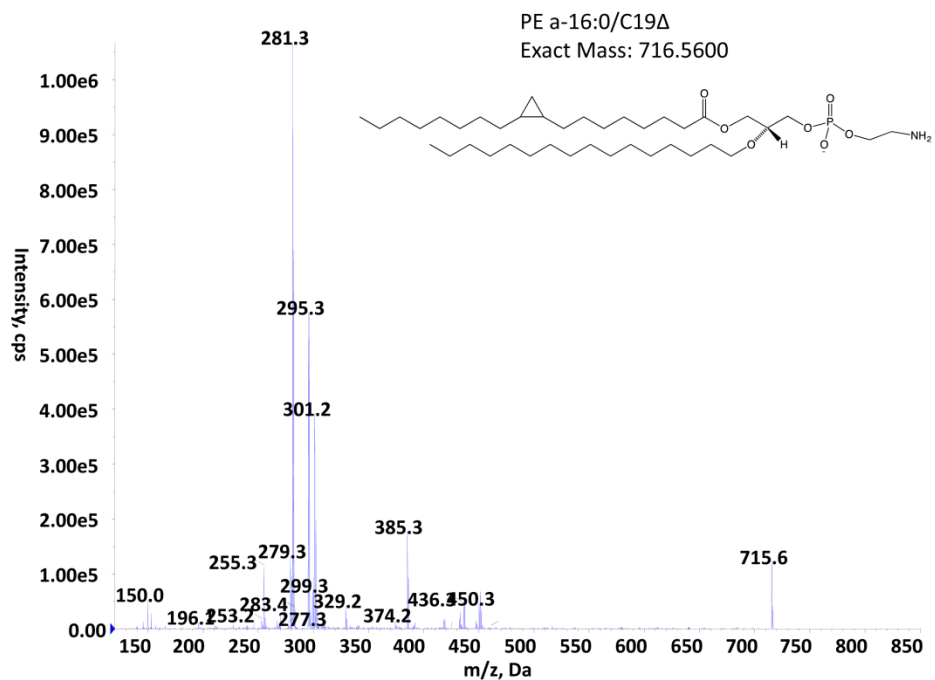

**C**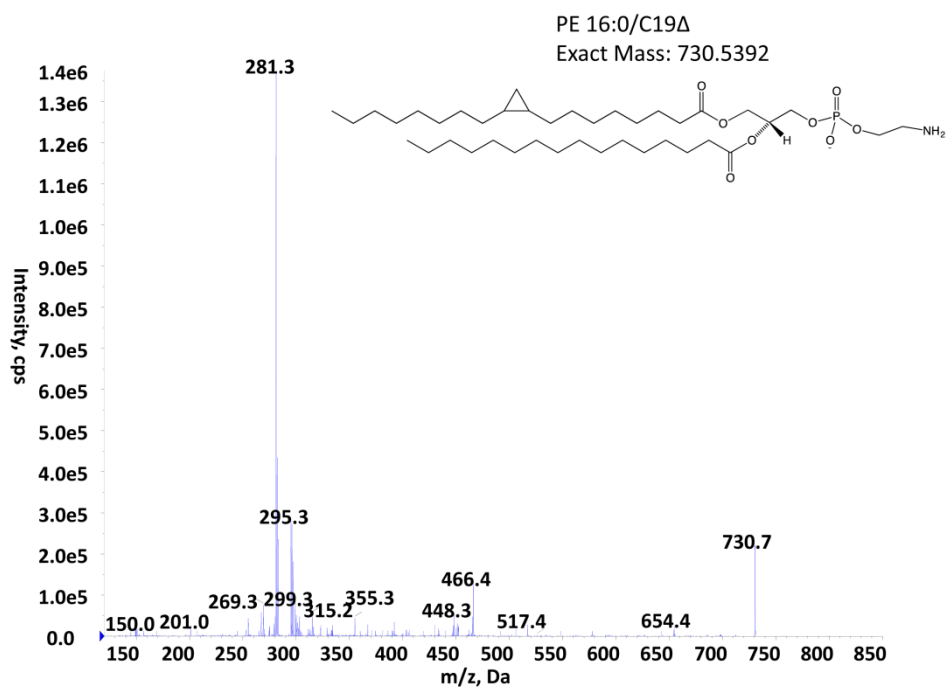**D**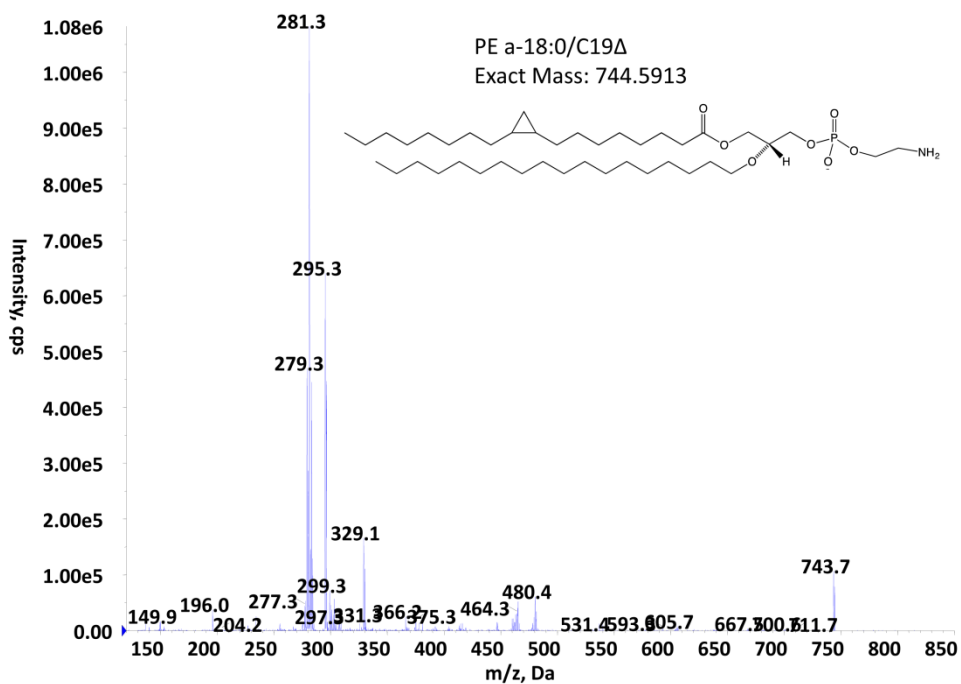

E

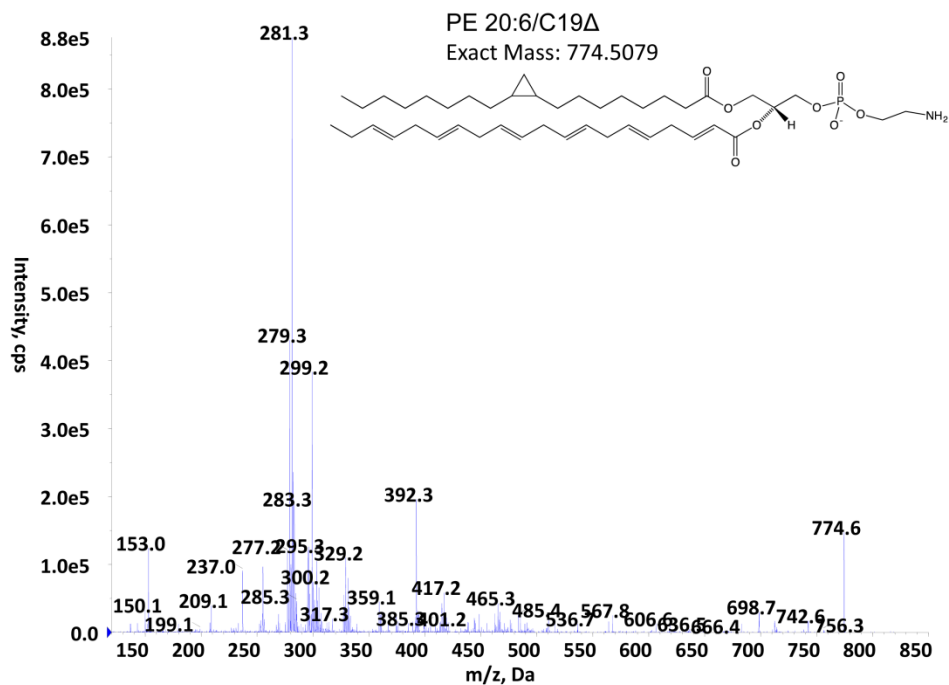

F

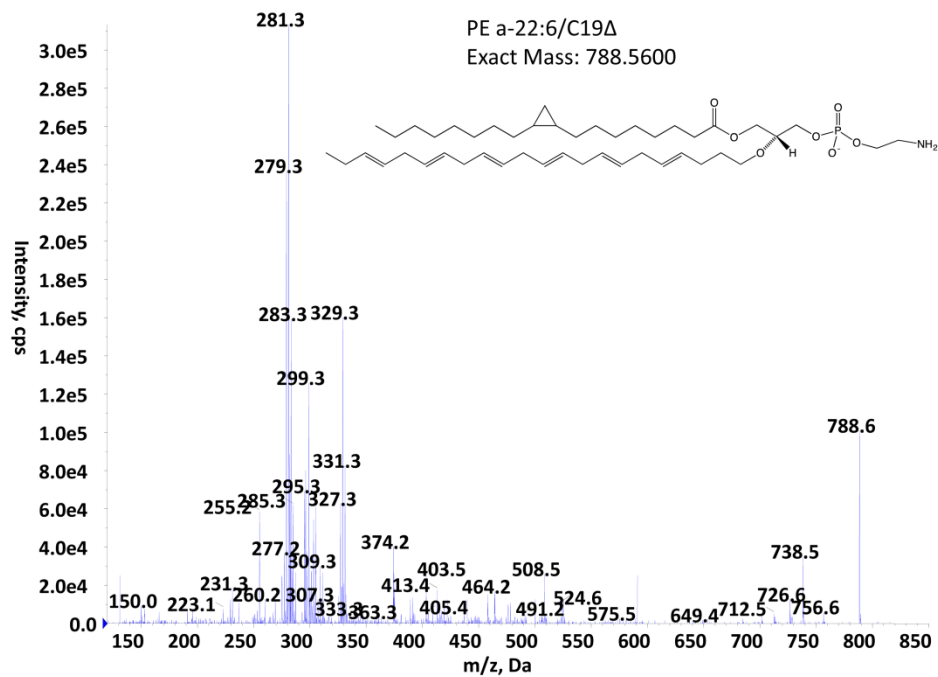

## G

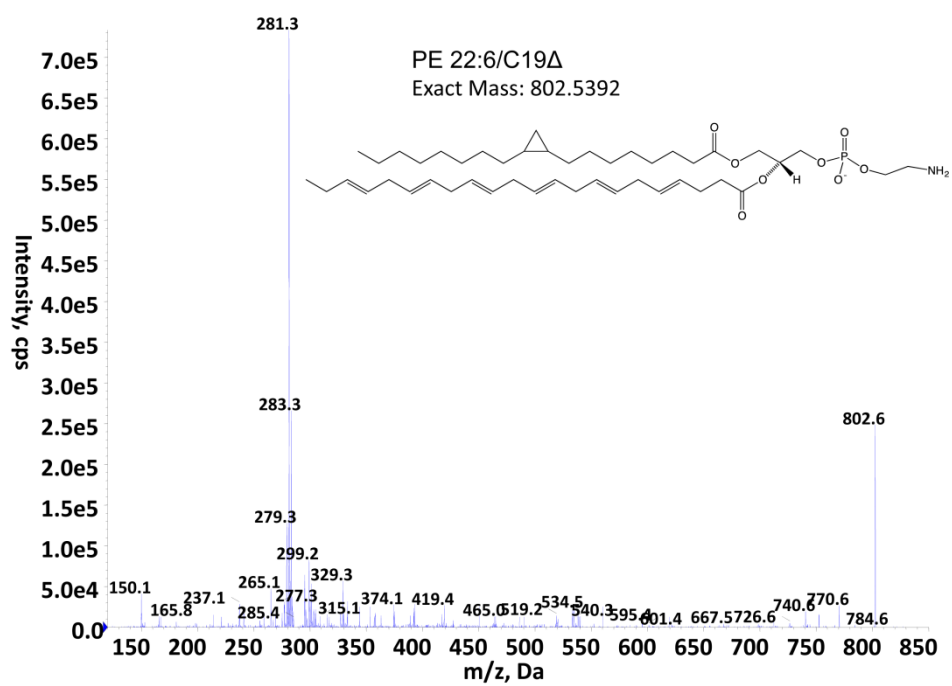

Supplement: S6 Fig — (A) ES-MS/MS parents of 295 m/z (C19Δ acyl fragment) in negative mode (600–1000 m/z). (B-G) are daughter ion ESI-MS/MS spectra of ions identified in S6A Fig: 716, 730, 744, 774, 788, 802 m/z respectively. (PDF) [file pntd.0005171.s006.pdf]

**A**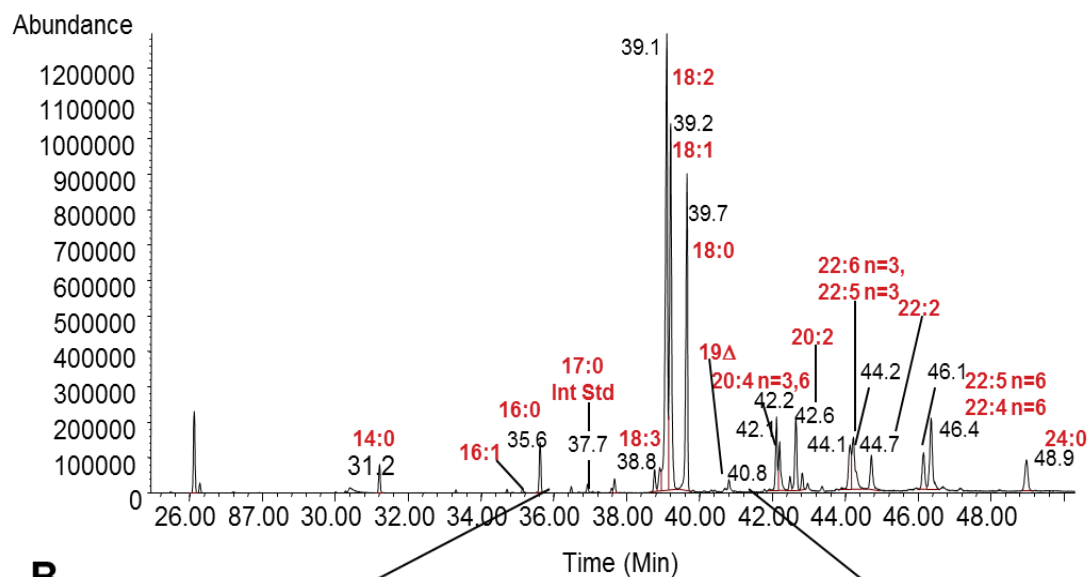**B**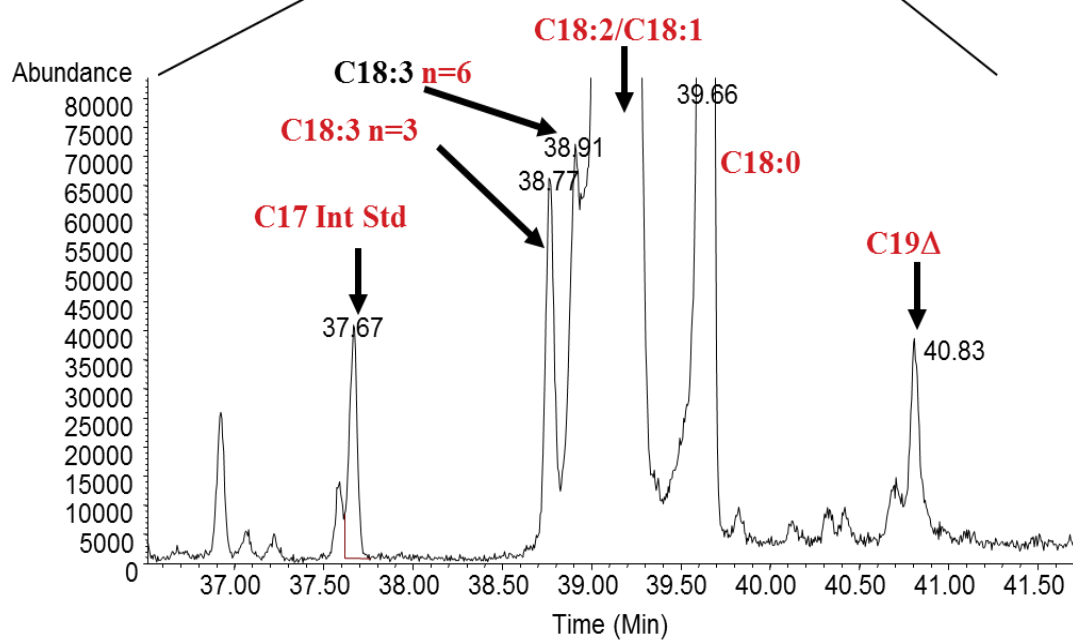

Supplement: S7 Fig — (A) Chromatogram including all the FAMES species with retention times spanning from 26.00 to 50.00 min for mid log phase parasites for each strain detailed in Table 1. (B) Magnification of the chromatogram for the identification of the spectral peak with a retention time of ∼40 min corresponding to C19Δ. The identity of C19Δ FAME was confirmed by retention time and spectral comparison with bacterial FAME standards, which includes C19Δ. (PDF) [file pntd.0005171.s007.pdf]

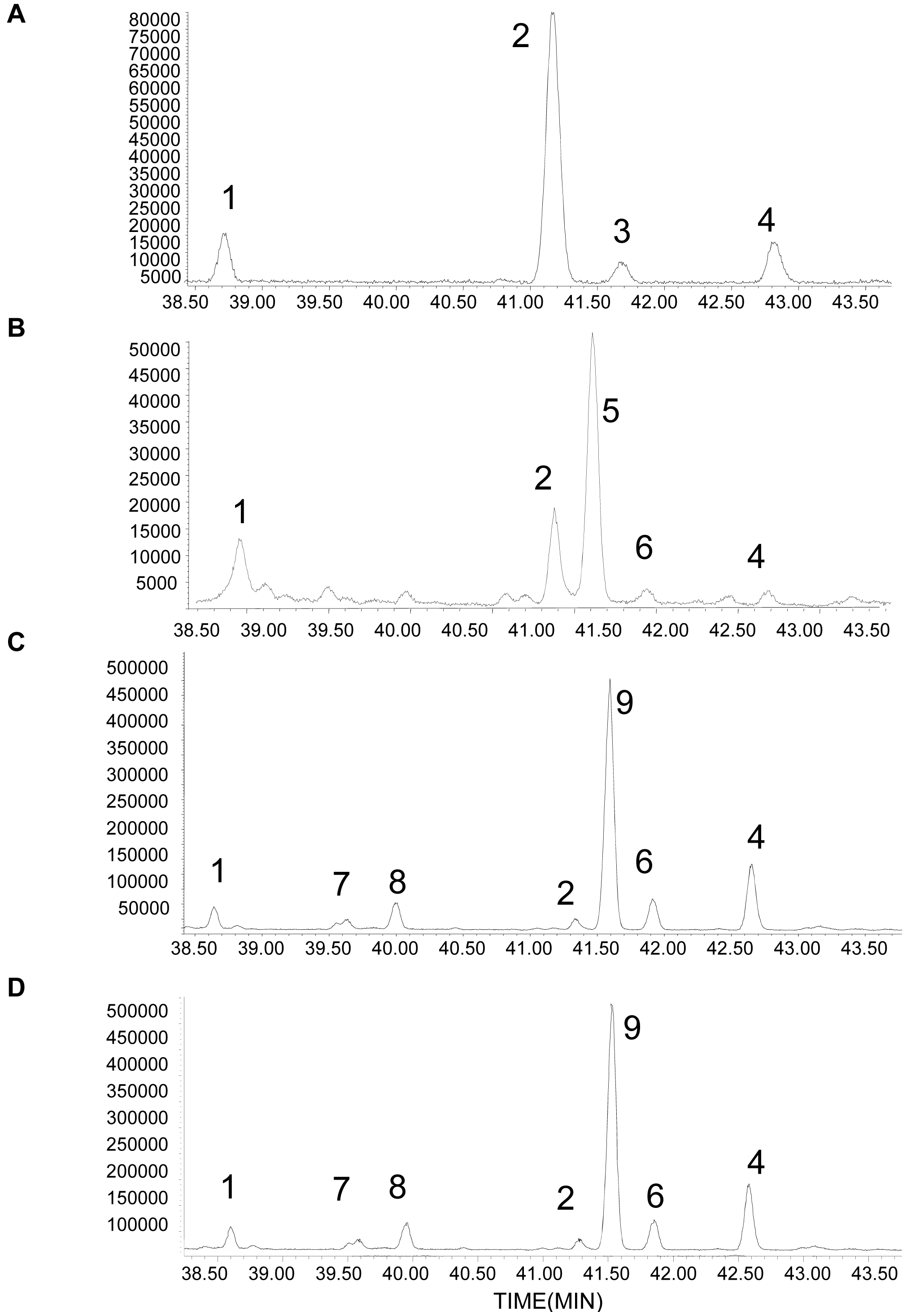

Supplement: S8 Fig — TIC of chromatogram 39.50–43.50 min for (A) Ldi263, (B) MF200.5, (C) AmB1000.1, (D) AmB1000.1+MT. Numbered peaks refer to Table 4 for identification. (TIF) [file pntd.0005171.s008.tif]
