# Supplementary material for: Different Mutations in a P-type ATPase Transporter in Leishmania Parasites are Associated with Cross-resistance to Two Leading Drugs by Distinct Mechanisms
Source: PLoS Negl Trop Dis. 2016 Dec 2;10(12):e0005171. doi: 10.1371/journal.pntd.0005171 (PMC5135041; doi:10.1371/journal.pntd.0005171)
Supplement: S1 Table — (PDF) [file pntd.0005171.s009.pdf]

Supplementary Table S1. Estimated ploidy for Ldi263AmB1000.1 and its WT parent (Ldi263WT).

	Ldi263WT		Ldi263AMB1000.1	
**Chr**	**Median FPKM**	**Ploidy Estimate**	**Median FPKM**	**Ploidy Estimate**
LinJ.01	26.5366	2	29.3728	2
LinJ.02	27.35685	2	29.54255	2
LinJ.03	26.77355	2	29.08575	2
LinJ.04	27.3013	2	29.5662	2
LinJ.05	28.1074	2	42.7873	3
LinJ.06	28.0202	2	29.7669	2
LinJ.07	26.3567	2	28.352	2
LinJ.08	26.52965	2	29.29835	2
LinJ.09	32.9111	2	29.34605	2
LinJ.10	27.037	2	29.8045	2
LinJ.11	27.1969	2	29.1277	2
LinJ.12	53.3895	4	28.8899	2
LinJ.13	39.3248	3	42.1333	3
LinJ.14	26.40235	2	28.21675	2
LinJ.15	26.61885	2	28.25005	2
LinJ.16	26.7811	2	13.8955	1
LinJ.17	26.6867	2	28.2293	2
LinJ.18	26.6362	2	27.4423	2
LinJ.19	26.6425	2	27.83285	2
LinJ.20	26.2234	2	27.2882	2
LinJ.21	26.61365	2	35.4369	3
LinJ.22	26.6853	2	28.062	2
LinJ.23	27.0422	2	36.7171	3
LinJ.24	26.2478	2	27.5238	2
LinJ.25	26.4474	2	27.92435	2
LinJ.26	27.2747	2	27.8175	2
LinJ.27	25.838	2	28.22375	2
LinJ.28	26.1999	2	28.3328	2
LinJ.29	26.0999	2	27.6516	2
LinJ.30	25.92495	2	28.1439	2
LinJ.31	63.6798	5	54.7806	4
LinJ.32	38.81885	3	41.50375	3
LinJ.33	25.84645	2	27.2527	2
LinJ.34	25.79285	2	27.9632	2
LinJ.35	25.8012	2	27.8109	2
LinJ.36	25.3979	2	27.4582	2

Median median FPKM Ldi263AMB1000.1 = 28.3328

Median median FPKM Ldi263WT = 26.6425
