# Supplementary material for: Different Mutations in a P-type ATPase Transporter in Leishmania Parasites are Associated with Cross-resistance to Two Leading Drugs by Distinct Mechanisms
Source: PLoS Negl Trop Dis. 2016 Dec 2;10(12):e0005171. doi: 10.1371/journal.pntd.0005171 (PMC5135041; doi:10.1371/journal.pntd.0005171)
Supplement: S2 Table — (PDF) [file pntd.0005171.s010.pdf]

Table S2

SNPs deduced from the sequencing of LdiAmB1000.1 and absent from the sequencing of its Ldi263WT parent.

SNP type	Total[1]	In CDS[1,2]	In CDS non-synonymous[1,2]
**Homozygous**	18	3	3
**Heterozygous**	470	123	85
**Total**	488	126	88

[1] Number of SNPs with ≤ 0.1% wrong call rates (phred scale quality value of ≥ 30).

[2] CDS, Coding sequence
