# Supplementary material for: Different Mutations in a P-type ATPase Transporter in Leishmania Parasites are Associated with Cross-resistance to Two Leading Drugs by Distinct Mechanisms
Source: PLoS Negl Trop Dis. 2016 Dec 2;10(12):e0005171. doi: 10.1371/journal.pntd.0005171 (PMC5135041; doi:10.1371/journal.pntd.0005171)
Supplement: S3 Table — (PDF) [file pntd.0005171.s011.pdf]

Table S3

Mass spectrometric analysis of phosphatidylethanolamine species containing cyclopropyl fatty acid in *Leishmania infantum.*

Nominal Mass (m/z)	Species	Observed Mass (m/z)	Theoretical Mass (m/z)	Delta
714	PE a-16:1/19Δ	714.545	714.5443	0.0007
716	PE a-16:0/19Δ	716.5654	716.5600	0.0054
728	PE 16:1/19Δ	728.5247	728.5236	0.0011
742	PE a-18:1/19Δ	742.5757	742.5756	0.0001
744	PE a-18:0/19Δ	744.5565	744.5913	0.0348
774	PE 20:6/19Δ	774.4994	774.5079	0.0085
776	PE 20:5/19Δ	776.5064	776.5236	0.0172
788	PE a-22:6/19Δ	788.5768	788.5600	0.0168
