# Supplementary material for: Different Mutations in a P-type ATPase Transporter in Leishmania Parasites are Associated with Cross-resistance to Two Leading Drugs by Distinct Mechanisms
Source: PLoS Negl Trop Dis. 2016 Dec 2;10(12):e0005171. doi: 10.1371/journal.pntd.0005171 (PMC5135041; doi:10.1371/journal.pntd.0005171)
Supplement: S1 Dataset — (PDF) [file pntd.0005171.s012.pdf]

SI Datasets

Dataset S1: InDels

GeneID	Pos in gene	Xsome	Pos in xsome	Seq in ref	Seq in AmB1000.1	Qual	Gene annotation
LinJ.04.0800	102	LinJ.04	310482	A	AG	96.73	hypothetical protein conserved
LinJ.10.0521	67	LinJ.10	220111	CG	C	232.74	hypothetical protein unknown function
LinJ.21.1780	4173	LinJ.21	621120	CCCGCTCGT	C	658.73	hypothetical protein conserved
LinJ.35.0370	567	LinJ.35	94402	G	GT	211.73	ATP-dependent DEAD-box RNA helicase putative
