# Supplementary material for: Different Mutations in a P-type ATPase Transporter in Leishmania Parasites are Associated with Cross-resistance to Two Leading Drugs by Distinct Mechanisms
Source: PLoS Negl Trop Dis. 2016 Dec 2;10(12):e0005171. doi: 10.1371/journal.pntd.0005171 (PMC5135041; doi:10.1371/journal.pntd.0005171)
Supplement: S2 Dataset — (PDF) [file pntd.0005171.s013.pdf]

Dataset S2: AmB_SNPs-InCDS-NoSyn-Homozygous

GeneID	Pos in Gene	Xsome	Pos on xsome	Nt in ref	Nt in AmB1000.1	Qual	Codon in ref	Codon in Amb1000.1	AA in ref	AA in AmB1000.1	Gene annotation
LinJ.13.1590	1297	LinJ.13	619316	C	T	661.77	GGC	AGC	G	S	phospholipid-transporting ATPase 1-like protein
LinJ.16.1240	2476	LinJ.16	465851	G	C	206.84	CAG	GAG	Q	E	hypothetical protein conserved
LinJ.35.0520	5302	LinJ.35	211854	G	A	40.74	GCC	ACC	A	T	proteophosphoglycan ppg4
