# Supplementary material for: Different Mutations in a P-type ATPase Transporter in Leishmania Parasites are Associated with Cross-resistance to Two Leading Drugs by Distinct Mechanisms
Source: PLoS Negl Trop Dis. 2016 Dec 2;10(12):e0005171. doi: 10.1371/journal.pntd.0005171 (PMC5135041; doi:10.1371/journal.pntd.0005171)
Supplement: S3 Dataset — (PDF) [file pntd.0005171.s014.pdf]

Dataset S3: AmB_SNPs-InCDS-NoSyn-Heterozygous

GeneID	Pos in Gene	Xsome	Pos on xsome	Nt in ref	Nt in AmB1000.1	Qual	Codon in ref	Codon in Amb1000.1	AA in ref	AA in AmB1000.1	Gene annotation
LinJ.01.0010	878	LinJ.01	4198	T	G	85.77	CAG	CCG	Q	P	hypothetical protein unknown function
LinJ.01.0010	958	LinJ.01	4118	C	A	66.77	GAC	TAC	D	Y	hypothetical protein unknown function
LinJ.01.0830	760	LinJ.01	253154	G	C	90.77	GTC	CTC	V	L	calcium potassium channel CAKC putative
LinJ.06.0880	9025	LinJ.06	376997	C	G	32.77	CCG	GCG	P	A	hypothetical protein conserved
LinJ.06.1050	288	LinJ.06	436335	C	A	30.77	CAC	CAA	H	Q	hypothetical protein conserved
LinJ.09.0501	6364	LinJ.09	182743	G	A	193.77	GCA	ACA	A	T	hypothetical protein conserved
LinJ.10.0340	722	LinJ.10	133336	G	T	33.77	CGG	CTG	R	L	hypothetical protein conserved
LinJ.10.0540	517	LinJ.10	227576	T	C	388.77	TGT	CGT	C	R	mitogen-activated protein kinase 3 putative map kinase 3 putative MPK3
LinJ.11.1210	1435	LinJ.11	491106	C	A	75.77	CTC	ATC	L	I	ATP-binding cassette protein subfamily A member 2 putative ABCA2
LinJ.12.0668	1754	LinJ.12	422724	G	A	48.77	GGC	GAC	G	D	surface antigen protein 2 putative
LinJ.12.0668	1849	LinJ.12	422819	G	A	35.77	GTG	ATG	V	M	surface antigen protein 2 putative
LinJ.12.0668	362	LinJ.12	421332	T	A	30.77	GTC	GAC	V	D	surface antigen protein 2 putative
LinJ.12.0669	949	LinJ.12	426274	T	C	118.77	TCG	CCG	S	P	hypothetical protein conserved
LinJ.12.0730	315	LinJ.12	480195	G	T	30.77	CAG	CAT	Q	H	NADH flavin oxidoreductase NADH oxidase putative
LinJ.14.1600	1793	LinJ.00	103151	C	G	46.77	GGC	GCC	G	A	kinesin K39 putative
LinJ.14.1600	1823	LinJ.00	103121	C	T	31.77	AGC	AAC	S	N	kinesin K39 putative
LinJ.15.0490	3995	LinJ.15	168711	A	G	49.77	AAA	AGA	K	R	hypothetical protein
LinJ.16.0250	890	LinJ.16	87209	A	G	32.77	CTG	CCG	L	P	Elongator-like Protein 3a putative
LinJ.16.0410	259	LinJ.16	146492	C	G	32.77	GCA	CCA	A	P	hypothetical protein conserved
LinJ.16.0510	320	LinJ.16	184608	T	A	30.77	TAC	TTC	Y	F	hypothetical protein unknown function
LinJ.16.0670	1130	LinJ.16	239764	G	C	30.77	CCC	CGC	P	R	hypothetical protein conserved
LinJ.16.0760	494	LinJ.16	272618	A	G	36.77	CTG	CCG	L	P	transaldolase putative
LinJ.16.0780	619	LinJ.16	297446	G	T	40.77	CTG	ATG	L	M	hypothetical protein conserved
LinJ.16.1660	1855	LinJ.16	652205	G	T	32.77	GCC	TCC	A	S	hypothetical protein conserved
LinJ.17.1090	8678	LinJ.17	462033	T	C	31.77	CTC	CCC	L	P	hypothetical protein conserved
LinJ.19.1690	2363	LinJ.19	714968	T	C	36.78	GTG	GCG	V	A	hypothetical protein
LinJ.20.1120	5222	LinJ.20	475596	A	T	31.77	AAG	ATG	K	M	hypothetical protein conserved
LinJ.20.1190	167	LinJ.20	506449	A	G	139.77	AAC	AGC	N	S	endo-1 4-beta-xylanase z precursor-like protein
LinJ.20.1670	421	LinJ.20	700116	T	C	375.77	ATC	GTC	I	V	hypothetical protein conserved
LinJ.22.0180	780	LinJ.22	109657	C	A	31.77	GAG	GAT	E	D	hypothetical protein conserved
LinJ.22.0260	1640	LinJ.22	137353	T	C	30.77	GAT	GGT	D	G	hypothetical protein conserved
LinJ.22.0670	910	LinJ.22	299267	C	T	72.77	CCG	TCG	P	S	A2 protein
LinJ.22.0750	2161	LinJ.22	333116	C	G	30.77	CCG	GCG	P	A	hypothetical protein conserved
LinJ.22.1570	621	LinJ.00	118800	C	T	32.77	ATG	ATA	M	I	hypothetical protein
LinJ.24.0910	661	LinJ.24	318718	G	T	30.77	GGG	TGG	G	W	DNA polymerase theta polymerase domain only putative
LinJ.24.1310	388	LinJ.24	466251	C	T	103.77	CTC	TTC	L	F	amastin-like surface protein-like protein
LinJ.25.0810	190	LinJ.25	278762	C	G	33.77	CAG	GAG	Q	E	hypothetical protein unknown function
LinJ.26.0390	977	LinJ.26	106679	C	T	33.77	CGT	CAT	R	H	hypothetical protein conserved
LinJ.26.1840	1522	LinJ.26	671334	T	C	32.77	TCT	CCT	S	P	hypothetical protein conserved
LinJ.27.0020	373	LinJ.27	9735	C	T	37.77	GAG	AAG	E	K	hypothetical protein conserved
LinJ.28.0090	818	LinJ.28	32110	C	T	205.77	GCA	GTA	A	V	adenylate cyclase-like protein
LinJ.28.0650	1948	LinJ.28	239990	G	T	81.77	CTC	ATC	L	I	dynein heavy chain putative
LinJ.28.1830	3545	LinJ.28	666694	T	C	31.77	CAG	CGG	Q	R	hypothetical protein conserved
LinJ.28.2780	1534	LinJ.28	1015400	A	G	73.77	ATC	GTC	I	V	splicing factor 3B subunit 1 putative
LinJ.28.3080	101	LinJ.28	1100931	T	C	32.77	CAG	CGG	Q	R	hypothetical protein conserved
LinJ.29.1550	7243	LinJ.29	660421	C	T	36.77	GTG	ATG	V	M	phosphatidylinositol 4-kinase alpha putative
LinJ.29.2100	1474	LinJ.29	872953	T	A	33.77	AAC	TAC	N	Y	hypothetical protein conserved
LinJ.30.1260	2497	LinJ.30	408681	C	T	31.77	CGG	TGG	R	W	ubiquitin hydrolase putative cysteine peptidase Clan CA family C19 putative
LinJ.30.2390	67	LinJ.30	882511	G	A	37.77	CGC	TGC	R	C	hypothetical protein conserved
LinJ.30.3570	980	LinJ.30	1291683	C	A	51.77	CCG	CAG	P	Q	hypothetical protein conserved lorien protein
LinJ.30.3600	985	LinJ.30	1299262	C	T	32.77	CGA	TGA	R	*	hypothetical protein conserved
LinJ.31.0950	1715	LinJ.31	349667	C	A	85.77	CGT	CTT	R	L	sodium stibogluconate resistance protein putative
LinJ.31.2680	4168	LinJ.31	1251370	G	A	141.77	CGC	TGC	R	C	RNA polymerase ii largest subunit RPOIILS
LinJ.31.3400	1910	LinJ.00	27274	A	G	56.77	ATC	ACC	I	T	sodium stibogluconate resistance protein putative
LinJ.32.0230	530	LinJ.32	75959	A	G	33.78	CTC	CCC	L	P	hypothetical protein conserved
LinJ.32.3720	125	LinJ.32	1416369	C	G	435.77	TCC	TGC	S	C	hypothetical protein conserved
LinJ.33.0220	227	LinJ.33	63849	T	C	41.77	AAG	AGG	K	R	hypothetical protein unknown function
LinJ.33.0480	709	LinJ.33	157121	C	T	30.77	GAC	AAC	D	N	deoxyribodipyrimidine photolyase putative DNA repair enzyme putative
LinJ.33.0960	398	LinJ.33	317316	G	T	194.77	TGC	TTC	C	F	40S ribosomal protein S3 putative
LinJ.34.1150	521	LinJ.34	483613	G	C	73.77	CCG	CGG	P	R	amastin-like surface protein putative
LinJ.34.1150	528	LinJ.34	483606	G	C	54.77	GAC	GAG	D	E	amastin-like surface protein putative
LinJ.34.1290	3084	LinJ.34	538725	G	T	64.77	AAC	AAA	N	K	ATP-dependent RNA helicase-like protein
LinJ.34.1720	362	LinJ.34	725055	T	C	76.77	CAC	CGC	H	R	amastin-like surface protein putative
LinJ.34.2870	539	LinJ.34	1207079	C	T	37.77	CCA	CTA	P	L	hypothetical protein conserved
LinJ.34.3010	1576	LinJ.34	1260461	G	A	36.77	GCC	ACC	A	T	RNA editing associated helicase 2 putative REH2
LinJ.35.0490	10403	LinJ.35	160741	C	G	36.77	TCT	TGT	S	C	proteophosphoglycan ppg4
LinJ.35.0500	10520	LinJ.35	187748	C	G	81.77	CCT	CGT	P	R	proteophosphoglycan ppg3 putative
LinJ.35.0500	4666	LinJ.35	181894	G	T	43.77	GCG	TCG	A	S	proteophosphoglycan ppg3 putative
LinJ.35.0510	1349	LinJ.35	192213	G	A	39.77	CGC	CAC	R	H	proteophosphoglycan ppg4
LinJ.35.0510	1351	LinJ.35	192215	G	A	39.77	GCC	ACC	A	T	proteophosphoglycan ppg4
LinJ.35.0510	8044	LinJ.35	198908	C	T	68.77	CAG	TAG	Q	*	proteophosphoglycan ppg4
LinJ.35.0520	4873	LinJ.35	211425	G	C	45.77	GCC	CCC	A	P	proteophosphoglycan ppg4
LinJ.35.0520	4876	LinJ.35	211428	G	A	43.77	GTC	ATC	V	I	proteophosphoglycan ppg4
LinJ.35.0530	499	LinJ.35	218260	C	T	117.77	CCA	TCA	P	S	proteophosphoglycan 5
LinJ.35.0530	5953	LinJ.35	223714	G	A	43.77	GTC	ATC	V	I	proteophosphoglycan 5
LinJ.35.0940	122	LinJ.35	394424	G	A	34.77	GGG	GAG	G	E	hypothetical protein conserved
LinJ.35.1140	780	LinJ.35	496132	G	T	41.77	TGG	TGT	W	C	oligosaccharyl transferase-like protein
LinJ.35.1170	741	LinJ.35	512202	G	T	36.77	TGG	TGT	W	C	oligosaccharyl transferase subunit putative
LinJ.35.1600	481	LinJ.35	669610	C	A	34.77	GAA	TAA	E	*	hypothetical protein conserved
LinJ.35.2300	2189	LinJ.35	909995	C	T	32.77	CCC	CTC	P	L	hypothetical protein conserved
LinJ.36.1730	263	LinJ.36	645706	G	T	32.77	CCG	CAG	P	Q	proteasome beta 5 subunit putative
LinJ.36.2510	541	LinJ.36	953835	T	C	316.77	TGC	CGC	C	R	sterol 24-c-methyltransferase putative
LinJ.36.2520	541	LinJ.36	957624	T	C	78.77	TGC	CGC	C	R	sterol 24-c-methyltransferase putative
LinJ.36.2540	397	LinJ.36	966698	G	A	144.77	GGC	AGC	G	S	hypothetical protein conserved
LinJ.36.4950	1437	LinJ.36	1824949	T	A	30.77	TTT	TTA	F	L	methionine synthase reductase mitochondrial precursor-like protein
